# Exploring the common mechanism of vascular dementia and inflammatory bowel disease: a bioinformatics-based study

**DOI:** 10.3389/fimmu.2024.1347415

**Published:** 2024-04-25

**Authors:** Yujiao Wang, Daojun Xie, Shijia Ma, Nan Shao, Xiaoyan Zhang, Xie Wang

**Affiliations:** ^1^ Anhui University of Chinese Medicine, Hefei, Anhui, China; ^2^ Encephalopathy Center, The First Affiliated Hospital of Anhui University of Chinese Medicine, Hefei, Anhui, China

**Keywords:** vascular dementia, inflammatory bowel disease, the brain-gut axis, pathogenesis, biomarkers, immune cells infiltration

## Abstract

**Objective:**

Emerging evidence has shown that gut diseases can regulate the development and function of the immune, metabolic, and nervous systems through dynamic bidirectional communication on the brain-gut axis. However, the specific mechanism of intestinal diseases and vascular dementia (VD) remains unclear. We designed this study especially, to further clarify the connection between VD and inflammatory bowel disease (IBD) from bioinformatics analyses.

**Methods:**

We downloaded Gene expression profiles for VD (GSE122063) and IBD (GSE47908, GSE179285) from the Gene Expression Omnibus (GEO) database. Then individual Gene Set Enrichment Analysis (GSEA) was used to confirm the connection between the two diseases respectively. The common differentially expressed genes (coDEGs) were identified, and the STRING database together with Cytoscape software were used to construct protein-protein interaction (PPI) network and core functional modules. We identified the hub genes by using the Cytohubba plugin. Gene Ontology (GO) and Kyoto Encyclopedia of Genes and Genomes (KEGG) pathway enrichment analysis were applied to identify pathways of coDEGs and hub genes. Subsequently, receiver operating characteristic (ROC) analysis was used to identify the diagnostic ability of these hub genes, and a training dataset was used to verify the expression levels of the hub genes. An alternative single-sample gene set enrichment (ssGSEA) algorithm was used to analyze immune cell infiltration between coDEGs and immune cells. Finally, the correlation between hub genes and immune cells was analyzed.

**Results:**

We screened 167 coDEGs. The main articles of coDEGs enrichment analysis focused on immune function. 8 shared hub genes were identified, including *PTPRC*, *ITGB2*, *CYBB*, *IL1B*, *TLR2*, *CASP1*, *IL10RA*, and *BTK*. The functional categories of hub genes enrichment analysis were mainly involved in the regulation of immune function and neuroinflammatory response. Compared to the healthy controls, abnormal infiltration of immune cells was found in VD and IBD. We also found the correlation between 8 shared hub genes and immune cells.

**Conclusions:**

This study suggests that IBD may be a new risk factor for VD. The 8 hub genes may predict the IBD complicated with VD. Immune-related coDEGS may be related to their association, which requires further research to prove.

## 1 Introduction

Vascular dementia (VD), the second major category of dementia, also known as vascular cognitive impairment (VCI), is mainly manifested as a progressive and fluctuating decline in memory, language, computation, visuospatial ability, attention, and power of execution (1). VD is caused by multiple factors, which can lead to neurovascular injury, further reducing cerebral blood flow, and cognitive dysfunction. Its main pathogenesis includes a neuroinflammatory response, oxidative stress, destruction of the blood-brain barrier (BBB), uncoupling of neurovascular units, destruction of mitochondrial morphology and function, demyelination and myelin injury, neurotoxicity, etc (2). The absence of well-established and universally accepted neuropathological criteria, along with the diversity and overlapping definitions of cerebrovascular lesions, pose challenges in determining the true prevalence of pure VCI in autopsy studies (3). However, the evidence has shown that the cognitive impairment of VD is more severe in comparison to other neurodegenerative diseases (4). An epidemiological investigation showed that there was significantly higher rate in hospitalization and mortality of VCI patients than those of non-VCI patients, similar to that of Alzheimer’s disease (AD) patients (5).

The evidence that intestinal diseases can affect nervous system function, such as depression, anxiety, dementia, Parkinson’s disease (PD), through the brain-gut axis (6), has been verified, and this relationship may be bidirectional (7–11). Inflammatory bowel disease (IBD), including crohn’s disease (CD) and ulcerative colitis (UC), is a chronic, relapsing, immune-mediated intestinal disease (6, 7). It is characterized by the overlapping effects of genetic mutations (12), environmental factors (13), intestinal microbiota disorders (14), and immune factors (15). The connection between IBD and the nervous system has been extensively studied. For example, a clinical research showed that the prevalence of anxiety and depression in IBD patients is 32.1% and 25.2% respectively (16). Similarly, patients with depression are more commonly diagnosed with IBD, and psychiatric symptoms are significantly associated with adverse disease activity-related outcomes in IBD (17). Furthermore, the relationship between IBD and PD is also bidirectional due to genetic overlap, abnormal transfer of α-synuclein from the gastrointestinal tract to the central nervous system (CNS), and neuroinflammatory responses. This connection has been corroborated by multiple studies and experiments (18–20). In addition, studies on dementia from the direction of the brain-gut axis involve many aspects, such as epidemiology, pathogenesis, and prognosis (21). For example, an epidemiological study showed that IBD might not only be a risk factor for dementia, but also lead to an earlier age of dementia. This was consistent with another study, revealing a positive association between IBD and dementia risk, particularly in patients with UC (8). In addition, a meta-study also uncovered an increased long-term risk of dementia in IBD patients (22). However, the association mechanism between IBD and dementia may be multifactor, which may be related to chronic inflammation, accelerated atherosclerosis, and hypercoagulability (23), especially thromboembolic events, thrombocytosis, hyperlipidemia, and hyperhomocysteinemia. All these factors may further aggravate the occurrence and development of VD (24). On the one hand, a study had shown that the gut microbes might affect neuroinflammatory responses through microglia (25). On the other hand, gut microbes could affect the generation ability of hippocampal neurons through metabolism, endocrine signaling, and nervous system driving mechanisms (26–28), thus affecting cognitive function. These findings uncover a fact that gut microbes can influence the development and impairment of cognitive function.

**Figure 1:**
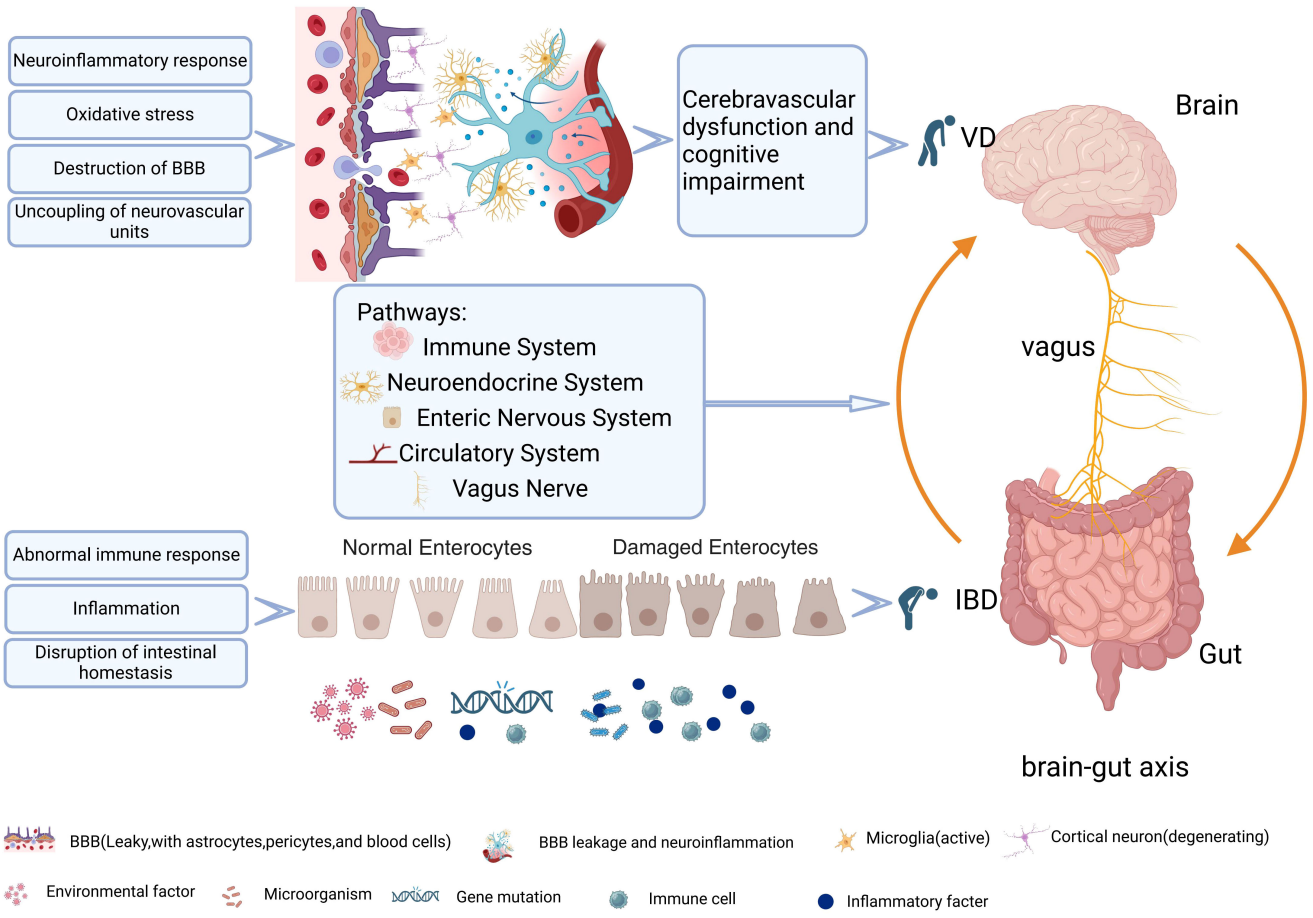
VD and IBD mechanism diagram and cerebroenteric axis pathway.

At present, the brain-gut axis not only connects the brain to the gut itself, but also represents the interaction between the brain-gut axis system and the central nervous system (CNS), endocrine chemical signaling system, immune regulation, microbiome, metabolism, and brain-gut barrier function (29) (**Figure 1**). Although extensive evidence has shown that gut diseases are closely associated with dementia, the underlying mechanisms have not been fully elucidated. In particular, limited research has been done to discover the relationship between VD and intestinal disease. Based on bioinformatics technology, this study explores the internal relationship between VD and IBD by means of searching for common differentially expressed genes (coDEGs) in VD brain tissue and UC colon tissue. The shared gene signatures identified are expected to elaborate the disease-related biological mechanism. The exploration of the common biological pathways can provide a new perspective for further study and treatment.

## 2 Materials and methods

**Figure 2:**
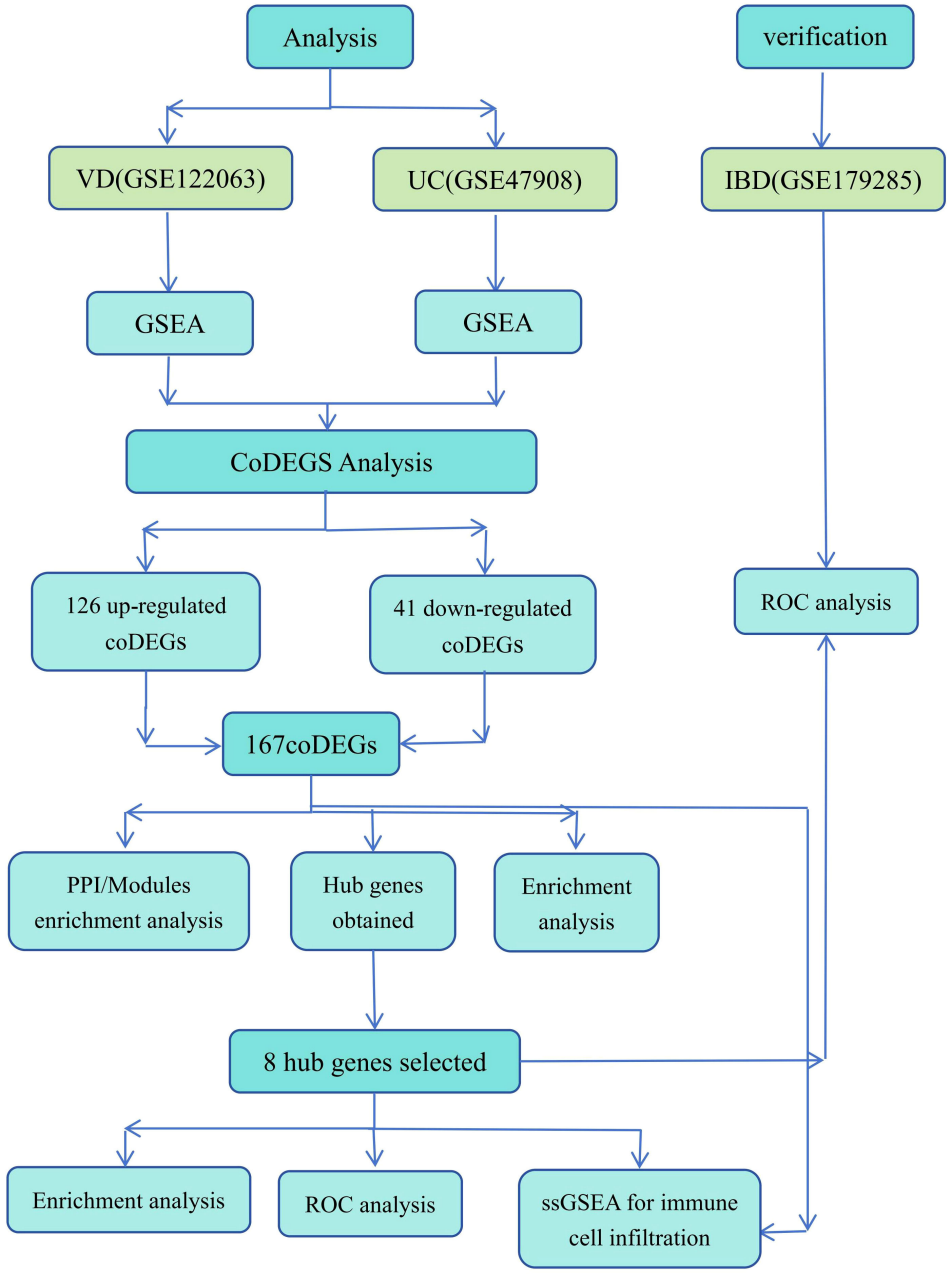
Flow chart of research design.

The overall design of the study is illustrated in **Figure 2**.

### 2.1 Data collection

Gene expressions of VD and UC were downloaded from the GEO (http://www.ncbi.nlm.nih.gov/geo/) database with the type of microarray. The independent datasets of GSE122063 for VD, including 36 tissues from 8 VD patients and 44 tissues from 11 healthy persons from hippocampal and frontal lobes, and GSE47908 for UC, including 19 UC tissues and 15 healthy tissues from human colonic biopsies were download for better investigating the mechanistic relationship between VD and UC. The UC validation datasets GSE179285 contained 70 inflammatory colon tissues from IBD, 153 non-inflammatory colon tissues from IBD, and 31 colon tissues from healthy controls. After obtaining the gene expression matrix, the platform information was used for gene annotation.

### 2.2 Gene set enrichment analysis in VD and UC

Based on the bioinformatics analysis tools in Xiantao Academic website (https://www.xiantaozi.com/), we used the ClusterProfiler package in *R* software to implement Gene Set Enrichment Analysis (GSEA) analysis. The two data sets were enriched with reference to gene set c2.cp. kegg.v2022.1.Hs.symbols.Gmt (KEGG Pathway Database) to determine the GSEA KEGG pathways of VD and UC. The ggplot2 package was constructed to perform the classical visualization of GSEA graphs for the enrichment analysis results.

### 2.3 Differential expression genes identification between VD and UC

Based on the bioinformatics analysis tools in Xiantao Academic website, we used the package “limma” by implementing the *R* software to normalize the data obtained from GEO. Statistically differentially expressed genes (DEGs) were defined with adjusted *p*-value < 0.05 (*P*.adj < 0.05) and |log2 (fold change)| > 0.58 using limma package. The up-regulated and down-regulated DEGs of VD and UC were identified respectively using the limma package in *R*. The DEGs were visualized by volcano plots and heatmaps respectively. The ggplot2 package were used to generate the volcano plots of DEGs for VD and UC. Heatmaps were depicted using the ComplexHeatmap packages. Genes that were up-regulated or down-regulated in both VD and UC were respectively considered as up-coDEGs and down-coDEGs. The overlapped DEGs in the two datasets were detected using the VennDiagram *R* package.

### 2.4 Functional enrichment analysis

Gene Ontology (GO) and Kyoto Encyclopedia of Genes and Genomes (KEGG) enrichment analysis for the selected coDEGs was carried out by using the *R* ClusterProfiler package. After ID conversion of the input molecule list, enrichment analysis was performed using the ClusterProfiler package, and the top 10 main pathways of biological processes (BP), cellular components (CC) and KEGG were visualized. We defined the threshold set for the coDEGs with *FDR* (*q* value) < 0.01 and *P*.adj < 0.05 as statistically significant.

### 2.5 Construction of protein-protein interaction network, acquisition of key modules and hub genes

The STRING database (https://string-db.org/) was used to search for molecular interactions and predict protein interactions. The PPI network of 167 coDEGs in VD and UC was constructed, and the reference value was set to confidence score > 0.4. Cytoscape (version 3.9.1) software was used to further visualize the PPI network diagram. The molecular complex detection (MCODE) plugin in Cytoscope was used to pick out PPI interacting core gene clusters in coDEGs. The screening criteria for key modules were MCODE score > 5, degree cut-off = 2, node score cut-off = 0.2, Max depth = 100, k-score = 2. By using the CytoHubba plugin of Cytoscape software, the top 10 key genes were selected from 167 coDEGs by applying the maximum cross correlation (MCC) algorithm, maximum neighborhood component (MNC) algorithm, Degree algorithm, and edge permeability component (EPC) algorithm. Then, the intersection of the VennDiagram *R* package was used to screen out the hub genes.

### 2.6 Enrichment analysis, verification of expression level, and diagnostic value of hub genes

The GO and KEGG enrichment analysis of hub genes was performed using the ClusterProfiler package, and the results were visualized using the ggplot2 package. We used the qROC package in *R* software to draw a receiver operating characteristic (ROC) curve to evaluate the diagnostic value of the hub genes. We used the area under the curve (AUC) with 95% confidence intervals (CI) to assess the levels of hub genes respectively on VD and UC. In addition, the validation datasets GSE179285 were used for external verification of the shared hub genes, and the Mann-Whitney U test (Wilcoxon rank sum test) test was performed on hub genes according to the expression profile data characteristics to further verify the expression level of diagnostic biomarker genes.

### 2.7 Immune cell infiltration analysis

The immune infiltration analysis was performed by single sample gene set enrichment analysis (ssGSEA) in the GSVA package for further verifying the results of bioinformatics analysis and exploring the potential relationship between the two Diseases. The background gene sets of 782 marker genes in 28 kinds of immune cells were constructed. The infiltration levels of VD and UC gene sets in 28 kinds of immune cells were calculated, and the infiltration levels of 16 kinds of immune cells were visualized. Combined with the data analysis of immune cells and immune functions, the heatmap software package was applied to draw the clustering heatmap of data sets and immune cells to show the infiltration degree of different immune cells in different samples. The ggplot2 package in *R* was used to draw a group box plot to compare the expression of different types of immune cells in the samples of the disease group and the control group. Meanwhile, the T test or Wilcox rank sum test was used to compare the differences between the groups, and there was a statistical difference when *P* < 0.05.

Finally, we extracted the expression matrix of the hub gene from the gene expression matrix, combined it with our immune cell abundance matrix, calculated the correlation coefficient and *p*-value, defined *P* < 0.05 as statistically significant differences, drew the correlation heatmap of immune cells and hub gene, and used the heatmap software package in *R* software for visual analysis.

## 3 Results

### 3.1 GSEA analysis in VD and UC

**Figure 3:**
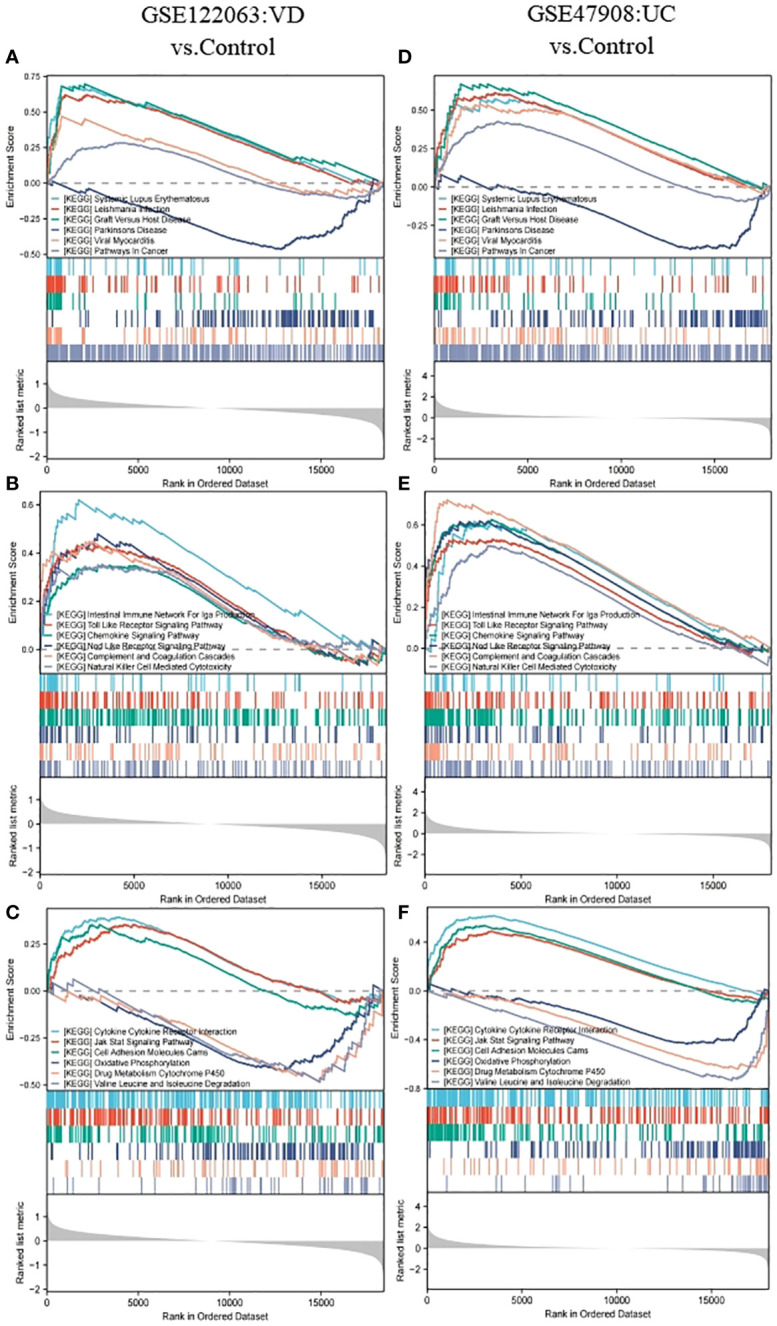
GSEA analysis for GSE122063 (VD) and GSE47908 (UC). **(A, D)** The genes of VD and UC were both enriched in terms of human diseases. **(B, E)** The genes of VD and UC were both enriched in terms of biological organic systems. **(C, F)** The genes of VD and UC were both enriched in terms of metabolism and environmental information processing.

The gene set of GSEA KEGG pathway reference MSigDB KEGG database (https://www.gsea-msigdb.org/gsea/msigdb/collections.jsp), *P*. adj < 0.05 and *FDR* < 0.25 was considered statistically significant. In VD, 40 pathways were enriched, while 46 pathways were in UC, and there were 18 intersection pathways between the two diseases. Interestingly, among these intersecting KEGG categories, immunization items accounted for the largest proportion 44.44%, including immune system pathways at 33.33 and human immune diseases at 11.11. Among the enriched pathways of human diseases, the common enrichment pathways included immune diseases with systemic lupus erythematosus and organ rejection disease, neurological degeneration with PD, and other diseases, including viral myocarditis, leishmaniasis, and tumor pathways. The pathways enriched in biological organic systems include IgA-producing intestinal immune network, chemokine signaling pathway, toll-like receptor signaling pathway, nod-like receptor signaling pathway, complement and coagulation cascade, as well as natural killer cell-mediated cytotoxicity, all belonging to the immune system. Pathways enriched for environmental information processing include cytokine-cytokine receptor interactions, cell adhesion molecules, and JAK-STAT signaling pathways. In metabolism, we also enriched three common significant pathways, including oxidative phosphorylation, drug metabolism-cytochrome P450, and degradation of valine, leucine, and isoleucine (**Figures 3A–F**).

### 3.2 Identification of differential expressed genes

**Figure 4:**
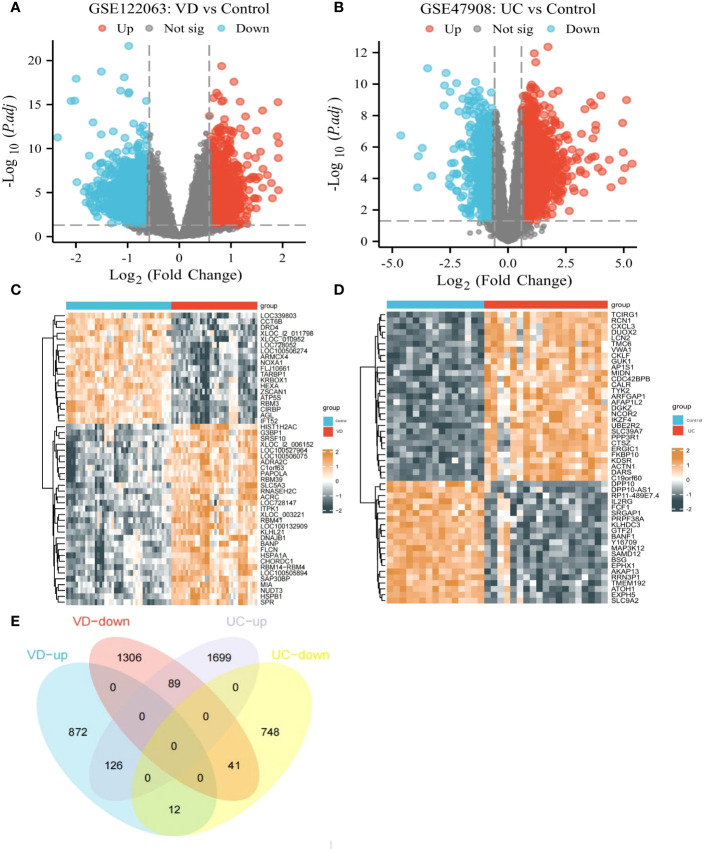
CoDEGs in VD and UC vs Control (healthy persons). **(A)** Volcano plots of DEGs from GSE122063. **(B)** Volcano plots of DEGs from GSE47908. **(C)** Heatmap of DEGs from GSE122063, including 36 tissues from VD and 44 tissues from healthy persons. **(D)** Heatmap of DEGs from GSE47908, including 19 UC tissues and 15 healthy tissues. **(E)** Venn diagram of co-DEGs extracted from DEGs of GSE122063 and GSE47908.

Based on the GSE122063 datasets, 2446 DEGs were picked out between VD patients and healthy controls (1010 up-regulated DEGs, 1436 down-regulated DEGs) (**Figures 4A, C**). Based on the GSE47908 datasets, 2715 DEGs were distinguished from UC patients and healthy controls (1914 up-regulated DEGs, 801 down-regulated DEGs) (**Figures 4B, D**). Finally, we identified 167 co-DEGs in VD and UC, including 126 up-regulated coDEGs and 41 down-regulated coDEGs (**Figure 4E**). This suggests that there may be molecular mechanistic similarities between VD and UC.

### 3.3 Functional enrichment analysis of CoDEGs

In order to better explain the biological functions of the shared genes, GO and KEGG pathway annotations were used to describe and analyze the 167 coDEGs screened in the above steps. GO was used to identify the top 10 significantly different BP, CC, and MF entries. KEGG pathway annotation identified the top 10 significantly enriched pathways of our interest.

**Figure 5:**
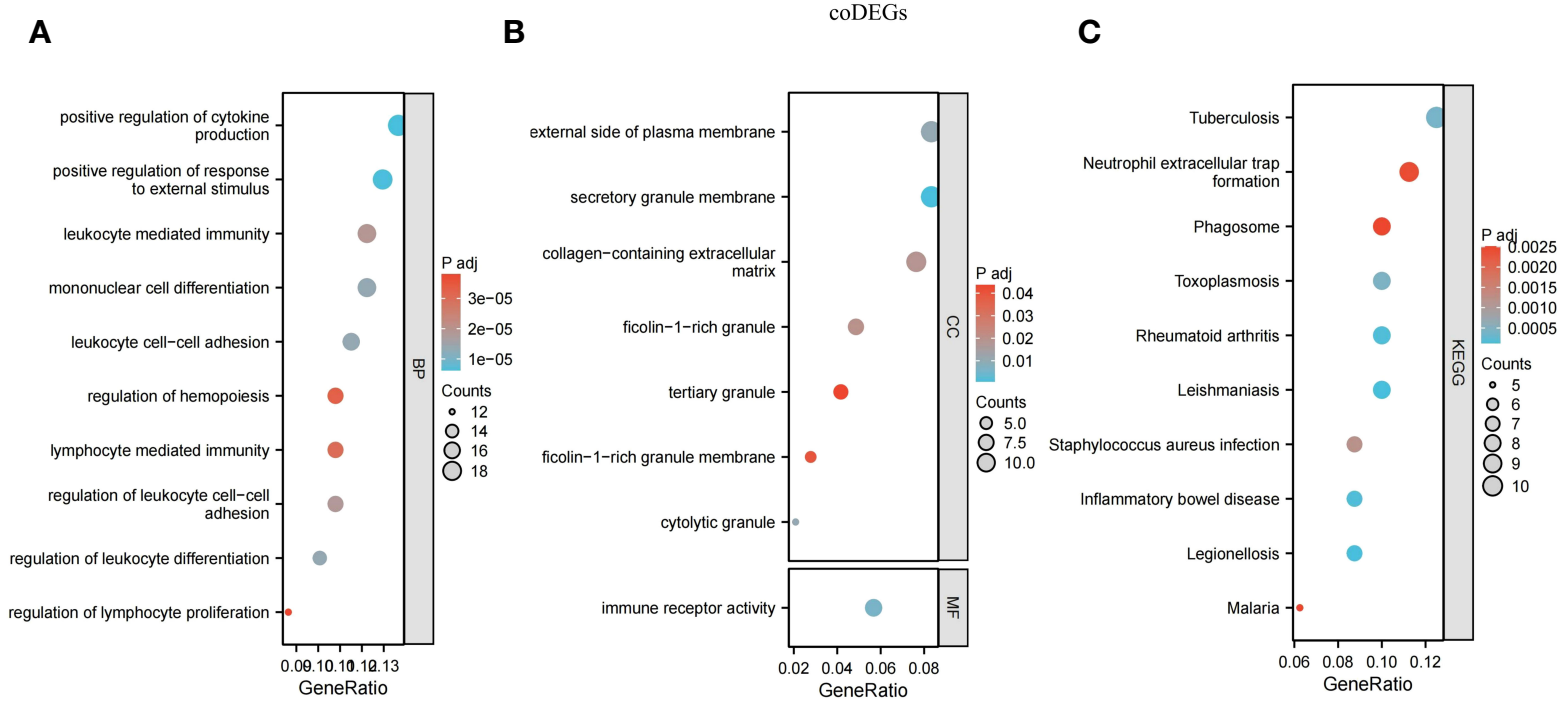
Functional annotation for coDEGs. **(A)** Biological process. **(B)** Cellular component and molecular function. **(C)** KEGG analysis. The size of the circle represents the number of genes enriched, and the larger the circle, the more genes enriched.

Many of the GO/KEGG entries we enriched were related to immunity. In the BP category, genes were mainly concentrated in positive regulation of cytokine production, positive regulation of response to external stimulus, mononuclear cell differentiation, leukocyte cell-cell adhesion, regulation of leukocyte differentiation, regulation of leukocyte cell-cell adhesion, leukocyte mediated immunity, lymphocyte-mediated immunity, regulation of hemopoiesis and regulation of lymphocyte proliferation (**Figure 5A**). For the CC ontology, they were mainly located in the secretory granular membrane, cell-dissolved particles, and the outer part of the plasma membrane, and immune receptor activity was mainly identified in MF analysis (**Figure 5B**). KEGG genes were enriched in human immune system diseases such as inflammatory bowel disease and rheumatoid arthritis, as well as infectious diseases such as leishmaniasis, legionellosis, and malaria, and in the organic immune system, neutrophil extracellular trap formation was enriched. Phagosomes in cellular processes were also identified (**Figure 5C**).

### 3.4 Construction of protein-protein interaction network of CoDEGs and acquisition of key gene modules

**Figure 6:**
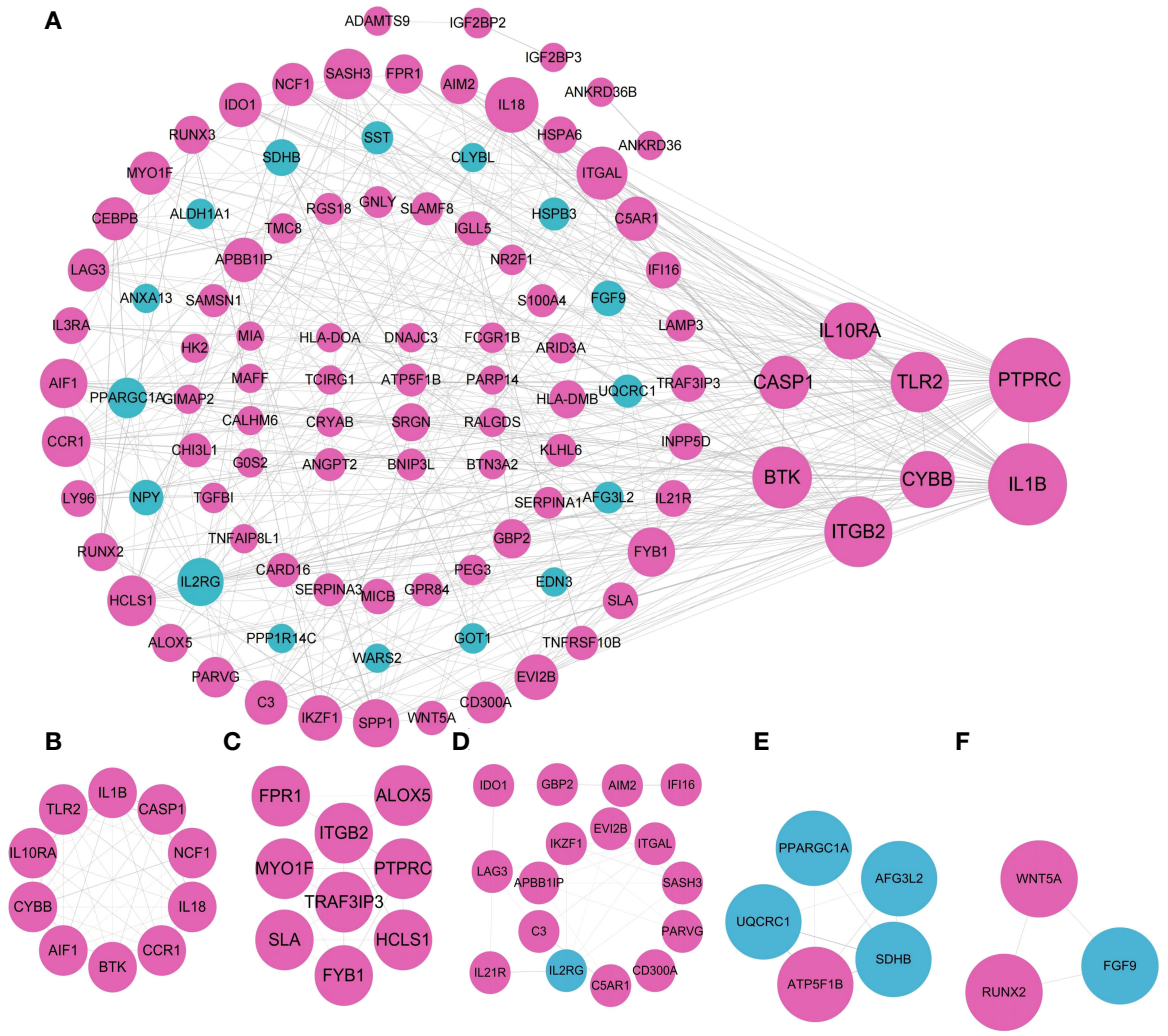
Visualization of the PPI network and the important modules. **(A)** PPI network. **(B–F)** submodule 1-5.

The 167 coDEGs analyzed in the above steps were located in the PPI interaction network to further explore their internal interactions in STRING database, with 157 nodes and 419 edges in total. 104 out of 167 genes were included in the network, including 85 out of 126 up-regulated genes and 19 out of 41 down-regulated genes. The functions of the rest of 63 genes have not yet been reported. Among them, purple nodes were up-regulated coDEGs, and blue nodes were down-regulated coDEGs. Node size was adjusted according to the value of connectivity. The higher the connectivity of nodes, the greater the weight of nodes in the PPI network diagram. As shown in **Figure 6A**, the top 10 gene with *CASP1*, *ITGAL*, *IL18*, *IL10RA*, *CYBB*, *TLR2*, *BTK*, *ITGB2*, *IL1B*, and *PTPRC* were picked out. Their weights were ≥20, all of which were up-regulated coDEGs. MCODE plug-in was used to screen and analyze the key modules of PPI. A total of 5 submodules were screened, and enrichment analysis was carried out in GO. Submodule 1 had 10 nodes and 41 edges (**Figure 6B**), belonging to *NADPH* oxidase complex, and its functions in GO enrichment analysis were as follows: positive regulation of helper T cell 1 factor, activation of the oxide synthesis process, active regulation of neuroinflammatory response, positive regulation of T-helper 1 cell cytokine production and reactive oxygen species biosynthetic process, positive regulation of granulocyte-macrophage colony-stimulating factor production). Submodule 2 had 9 nodes and 19 edges (**Figure 6C**), and the related functions were not enriched. Submodule 3 had 16 nodes and 31 edges (**Figure 6D**), which were related to the regulation of immune processes in GO enrichment analysis, including T cell activation, regulation of phagocytosis, and positive regulation of lymphocyte-mediated immunity. Submodule 4 had 5 nodes, and 8 edges (**Figure 6E**), GO enrichment analysis was related to respiratory electron transport chain, oxidative phosphorylation, and cellular respiration. Submodule 5 had 3 nodes and 3 edges (**Figure 6F**) and functions in the WikiPathway were related to osteoblast differentiation and related diseases.

### 3.5 Identification of hub genes by CytoHubba and functional enrichment analysis

**Figure 7:**
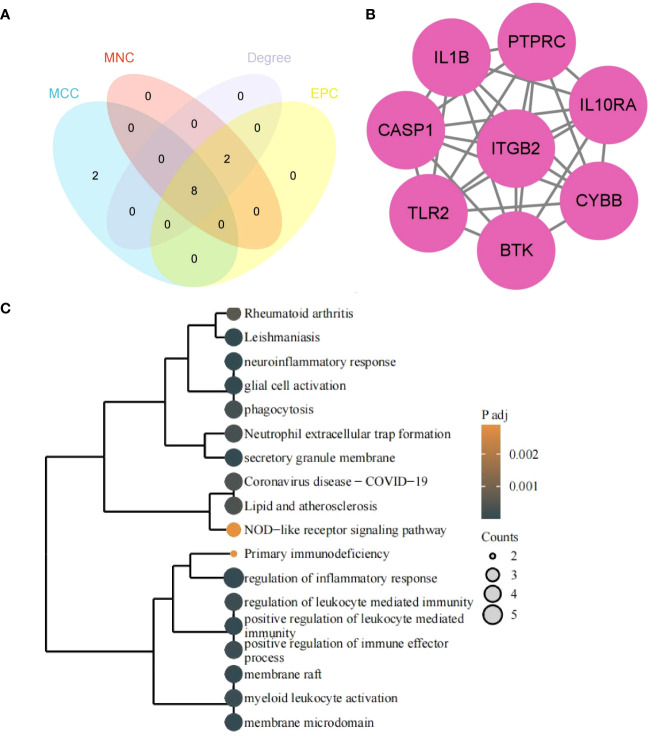
Hub genes and functional enrichment analysis. **(A)** Hub genes form four algorithms. **(B)** PPI network of hub genes. **(C)** GO/KEGG enrichment analysis of hub genes. The size of the circle represents the number of genes enriched, and the larger the circle, the more genes enriched.

The 8 hub genes were obtained via the CytoHubba plugin of Cytoscape software: *PTPRC*, *ITGB2*, *CYBB*, *IL1B*, *TLR2*, *CASP1*, *IL10RA*, *BTK* (**Figures 7A, B**). GO/KEGG enrichment analysis of hub genes suggested that the biological processes (BP) mainly focused on the immune and inflammatory responses mediated by leukocytes, neuroinflammatory response, glial cell activation and regulation of inflammatory response, bone marrow leukocyte activation, and regulation of immune effectors. The pathways enriched in KEGG mainly included leishmaniosis, lipid and atherosclerosis, rheumatoid arthritis in the human disease group, and the obvious immune system pathways enriched in neutrophil extracellular trap formation, NOD-like receptor signaling pathway, and primary immunodeficiency disease (**Figure 7C**).

### 3.6 Validation of hub genes

**Figure 8:**
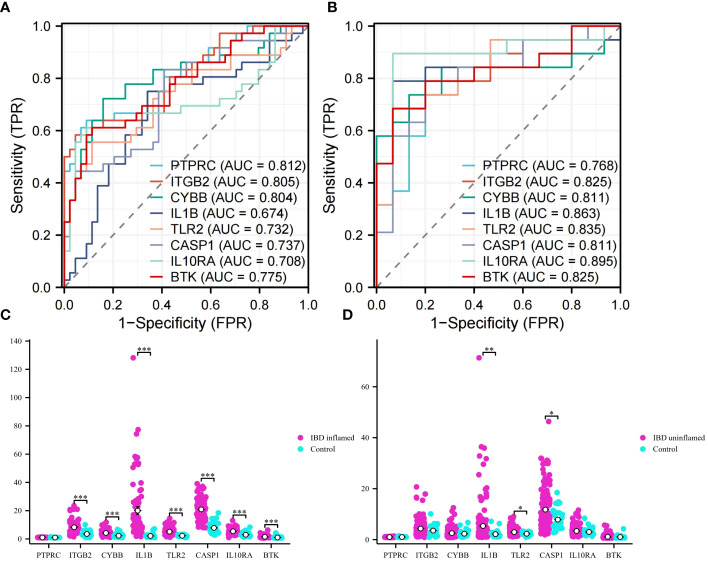
ROC analysis and validation. **(A)** ROC curves of hub genes in VD. **(B)** ROC curves of hub genes in UC. **(C)** Validation of expression levels in IBD inflammation datasets. **(D)** Validation of expression levels in IBM no inflammation dataset. This is a statistically significant marker that *P<0.05, **P<0.01, and ***P<0.001.

ROC analyses were performed for VD and UC respectively to evaluate the ability of diagnosis of the 8 hub genes. In the area under the curve (AUC) of 8 hub genes used to discriminate patients from healthy controls in VD datasets, the AUC area was greater than 0.674 (**Figure 8A**). But in UC datasets, they were greater than 0.768 (**Figure 8B**). The ROC curves recommended that the 8 hub genes had the ability to predict the risk of UC with VD. The results provide a basis for the development of novel targeted therapies for VD and UC diseases with these hub genes.

To obtain more accurate and reliable results, the UC external validation datasets were applied to verify the expression levels of 8 hub genes. It was found that the expression levels of genes *ITGB2*, *CYBB*, *IL1B*, *TLR2*, *CASP1*, *IL10RA*, and *BTK* in the samples of the IBD inflammation datasets were significantly higher than those in the healthy control (**Figure 8C**, *P* < 0.01). The results showed that these hub genes might have high diagnostic ability as characteristic biomarkers to predict VD in IBD patients. In addition, we verified the unaffected colon tissues of IBD patients in the validation datasets, and the results showed that *IL1B*, *TLR2*, and *CASP1* genes had early diagnostic effects (**Figure 8D**, *P* < 0.05). Due to the lack of training datasets for VD, we had to abandon the validation on the VD datasets.

### 3.7 Assessment and visual analysis of the immune infiltration

**Figure 9:**
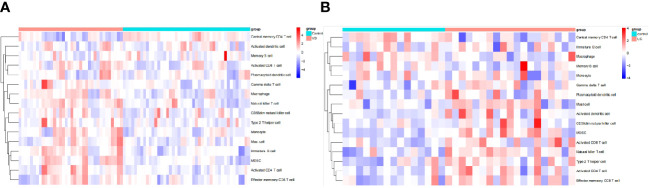
Immune cell infiltration analysis in VD and UC. **(A)** Hierarchical clustering of the distribution of the 16 immune cells in VD. **(B)** Hierarchical clustering of the distribution of the 16 immune cells in UC.

**Figure 10:**
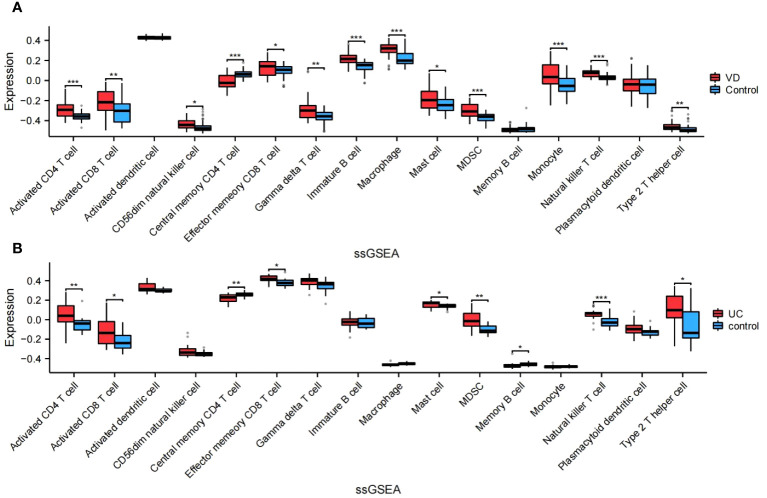
Relative proportions of the relative infiltration levels in VD and UC. **(A)** Relative proportions in VD. **(B)** Relative proportions in UC. This is a statistically significant marker that *P<0.05, **P<0.01, and ***P<0.001.

**Figure 11:**
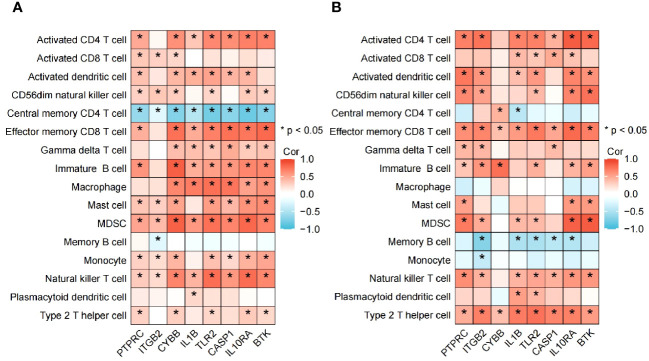
Association between the hub genes and immune cell infiltrationin VD and UC. **(A)** Heatmap of correlation analysis in VD. **(B)** Heatmap of correlation analysis in UC.

The algorithm of ssGSEA was used to quantify the distribution (**Figures 9A, B**) and relative proportions (**Figures 10A, B**) of the relative infiltration levels of 16 immune cells picked out from 28 immune cells in the GSE122063 and GSE47908 datasets. The correlation between the 8 hub genes and immune cell infiltration was analyzed (**Figures 11A, B**). The results could help us to better evaluate the connection about the immune pathways for the brain-gut axis between diseases and healthy controls.

There has a significant difference in the distribution and proportion of multiple immune cells in VD and UC patients and healthy controls. Including activated CD4 T cell, activated CD8 T cell, CD56dim natural killer cell, effector memory CD8 T cell, gamma delta T cell, immature B cell, macrophage, mast cell, MDSC, monocyte, natural killer T cell, and type 2 T helper cell were up-regulated in VD. Central memory CD4 T cell was down-regulated in VD. There was no significant difference between activated dendritic cells, memory B cells, and plasmacytoid dendritic cells. The up-regulated immune cells in UC were activated CD4 T cell, effector memory CD8 T cell, activated CD8 T cell, mast cell, MDSC, memory B cell, natural killer (NK) T cell, type 2 T helper cell. The down-regulated immune cells in UC included central memory CD4 T cells, which were statistically different. There was no statistically significant in activated dendritic cells, CD56dim NK cells, gamma delta T cells, immature B cells, macrophages, Monocytes, and plasmacytoid dendritic cells.

Biomarkers of 8 hub genes and infiltration analysis of 28 immune cells suggested that in VD gene concentration, central memory CD4 T cells are significantly negatively correlated with all hub genes (*P* < 0.05), while MDSC and NK T cells are positively correlated with all hub genes (*P* < 0.05), indicating that hub genes have a good level of infiltration with immune cells in VD.

In UC gene concentration, all hub genes were significantly negatively correlated with memory B cell, particularly in *ITGB2*, *IL1B*, *TLR2*, *CASP1*, and *IL10RA* (*P* < 0.05), while positively correlated with effector memory CD8 T cell and type 2 T helper cell (*P* < 0.05). These results provide key immune pathway evidence for the link between IBD and VD, and further demonstrate the feasibility of studying the co-pathogenesis and drug intervention targets of the two from the direction of the brain-gut axis.

## 4 Discussion

Vascular dementia is considered to be a disease that can be prevented and controlled. However, improvements in cognition have not been satisfactory in clinical trials, so medical practitioners have been focusing on innovation in vascular dementia mechanisms, risk factors, and new treatment options. Gut microbiota was considered to be an important influencing factor for stroke, and the bidirectional interaction between intestinal diseases and neurological diseases was still not clear. Integrative bioinformatics analyses are becoming more common in the exploration of new genes, potential diagnostic and prognostic biomarkers, underlying mechanisms, and potential therapeutic targets using big data. This approach can provide valuable insights into various diseases (30, 31).

The brain-gut axis is a bidirectional communication pathway. And all the central, intestinal, autonomic nervous systems, as well as the hypo-pituitary-adrenal (HPA) axis constitute a complete brain-gut axis system. The vagus nerve, immune and neuroendocrine systems, neurotransmitters and metabolites, and gut microbiota are the key nodes that play their respective mechanism effects in the brain-gut axis pathway (32–34). After decades of research by scientists, they have demonstrated a strong connection between the gut and the brain in mechanisms such as neurons, neurotransmitters, hormones, and immune-mediated regulation (35, 36). Whole-gene expression data may give us a hand to better understand the specific pathological links and provide molecular basis for brain-gut axis system. Firstly, 18 common pathways between GSE122063 (VD) and GSE47908 (UC) were shown by using he independent GSEA. Interestingly, the common pathways of both were highly clustered, such as mainly immune diseases and neurodegenerative diseases involved in human diseases, the common biological and organic systems belong to the immune system, and a considerable proportion of environmental information processing pathways, and metabolic pathways, suggesting that there might be a pathogenic correlation between VD and IBD. Next, GO and KEGG enrichment analyses were performed on 167 selected coDEGs. The main pathways of GO: BP function was mainly concentrated in the immune-mediated by leukocyte cells, monocytes, and lymphocytes, and the KEGG function enrichment was mainly concentrated in immune diseases, infectious disease, the immune system, and environmental information processing pathways. This suggested that the mechanism of connection between the two diseases might be related to immune and inflammatory regulation and environmental information processing. Interestingly, in a basic study (37, 38), scientists build a new microbiota-gut-brain axis, indicating that effector T cells migrate from the gut to the brain, specifically localizing in the leptomeninges. This migration enhances ischemic neuroinflammation by secreting interleukin-17 (IL-17), which in turn increases chemokine production in the brain parenchyma and leads to the infiltration of cytotoxic immune cells such as neutrophils. Furthermore, it had been well documented that inflammatory factors can lead to neuronal death resulting in cognitive and emotional dysfunction. In addition, in the study on the impact of ischemic stroke on the gut, cerebral ischemia could quickly cause intestinal ischemia, leading to a disruption in intestinal flora through free radical reactions, and enhance systemic inflammation and aggravate cerebral infarction. Transplantation of primary Intestinal Epithelial Stem Cells (IESCs) from healthy donors into post-stroke models could prevent depression-like behavior and cognitive impairment (39).

To further detect the coDEGs, 8 shared hub genes were selected, and GO/KEGG enrichment analysis was performed on the 8 shared genes. The GO: BP pathway mainly focused on the activation of glial cells, neuroinflammatory response and immune cell regulation, which was consistent with the results of previous studies (38). After verifying the functions of hub genes, we verified them in the IBD validation datasets, which showed the important role of 7 shared hub genes (*ITGB2*, *CYBB*, *IL1B*, *TLR2*, *CASP1*, *IL10RA*, and *BTK*) in IBD patients with VD and genes *IL1B*, *TLR2*, and *CASP1* might play an early diagnostic role in IBD complicated with VD. Because gut microbes can directly affect the characteristics of the immune system, immune activation might be the pathway through which gut microbes act on the CNS. Finally, 167 coDEGs were analyzed for immune infiltration and immune correlation, and 8 hub genes were analyzed for immune correlation. The results further confirmed the association between VD and IBD between coDEGs and hub genes and the existence of 16 immune cells in immune activities. This suggests that inflammatory diseases of the gut may affect central nervous system function through a specific immune system, especially in relation to vascular dementia, but of course, there are other factors involved, which need to be further studied.

*ITGB2* is predominantly expressed in immune cells and plays a crucial role in various metabolic pathways and immune functions, including leukocyte extravasation (40). *ITGB2* plays a critical role in T-cell development and function. Research has also associated *ITGB2* with chronic colitis (41, 42). In addition, a study suggested that *Itgb2+* microglia were found to function as a time-specific multifunctional immunomodulatory subcluster during ischemia reperfusion injury (CIRI). It has been proved that *ITGB2* plays a role in the pathological changes of IBD (43, 44), but the specific mechanism remains to be further explored. *TLR2* plays a fundamental role in pathogen recognition and activation of innate immunity. Knockout of *TLR2* has been shown to prevent impaired cerebral blood flow in early diabetes and VCI by counteracting overperfusion in long-term diabetes (45). The expression of *TLR2* is crucial in enhancing barrier function during injury and preserving tight junctions (46, 47), and are crucial in IBD (48–50). Furthermore, neutrophil extracellular traps (NETs) activated by *TLR2* and *TLR4* have been shown to worsen colon tissue damage and promote thrombotic events with active inflammatory bowel disease (IBD) (50), resulting in stroke and VD. BTK plays a crucial role in B-cell development and functions as a key player in both innate and adaptive immunity (51). In AD brain tissue, *BTK* may improve cognition in AD patients by preventing microglia activation and synaptic loss. *BTK* deficiency has been shown in various experiments to lead to severe colitis. This mechanism includes the activation of the NLRP3 inflammasome due to *BTK* defects (52), as well as its important role in preserving gut immune balance and preventing inflammation through the regulation of T-cell polarization (53). However, the mechanism of *BTK* in VD remains to be further explored. *CYBB*, also known as NADPHoxidase 2 (NOX2), is involved in the generation of reactive oxygen species (ROS) during ischemic stroke (IS) and is found to be significantly increased (54, 55). As has been demonstrated in numerous studies, oxidative stress is an important mechanism of vascular dementia (56, 57). In addition, the inadequate ROS production results in an upregulation of nuclear factor kappa-B (NF-κB)-regulated inflammatory genes, leading to the over activation of NF-κB and inflammasome in phagocytes. This sustained activation triggers the continuous release of pro-inflammatory cytokines and contributes to inflammatory conditions, such as IBD (58). *CASP1* plays a pivotal role in cellular immunity as an initiator of the inflammatory response. *CASP1* initiates a pro-inflammatory reaction by cleaving the inflammatory cytokines IL1B and IL18, thus participating in various inflammatory processes (59–61). Additionally, *CASP1* triggers pyroptosis (62). *IL1B* (Interleukin 1 Beta) is a member of the interleukin 1 cytokine family, known as a pro-inflammatory cytokine that plays a critical role in inflammatory disorders (63) and is typically regulated by the NLRP3-caspase1 axis (64). A number of studies have shown that the NLRP3/CASP1/IL1B pathway can participate in neuronal apoptosis and autophagy by mediating oxidative stress and neuroinflammatory response (65–67) in VD, leading to impairment and aggravation of cognitive function. Moreover, in IBD, numerous studies have demonstrated the activation of the NLRP3/CASP1/IL1B axis, which plays a crucial role in immune response, hyperinflammatory response, and pyroptosis, contributing to the pathogenesis and progression of the disease (68–71). *IL10RA* is cell surface receptor for the cytokine IL10 that participates in IL10-mediated anti-inflammatory functions, limiting excessive tissue disruption caused by inflammation. *IL10RA* modulates the miR15a/16-1/IL10RA/AKT3 axis to reduce brain damage and improve cognition in VD models (72). Intestinal microbial dysbiosis caused by *IL10RA* dysfunction can lead to early-onset IBD (73, 74) and refractory IBD (75). *PTPRC* requires for T-cell activation through the antigen receptor. Although there is substantial evidence for the role of T cells in VD and IBD (76, 77), the mechanism of action of *PTPRC* in both remains unclear. Despite the identification of shared genes using bioinformatics methods, further research is required to elucidate the interactions and relationships between these molecular markers through the brain-gut axis. It is evident that these biological pathways of molecular markers are primarily focused on immunity, inflammation, oxidative stress, microglia activation, among others. However, these pathways do not fully capture the signal transduction mechanisms between them, highlighting the necessity for additional pathway studies from the perspective of the brain-gut axis.

Neuroinflammatory response is mainly mediated by cytokines, chemokines, reactive oxygen species free radical transmitters, which are mainly secreted by microglia, astrocytes, endothelial cells (ECs) and peripheral derived immune cells. This response can be can be triggered by various damaging events, including hypoxia, ischemia, and infection (78). In normal organisms, neuroinflammation can maintain the stability of the immune microenvironment. However, it may also damage CNS due to excessive activation of the inflammatory response (79, 80), leading to cell injury, blood-brain barrier destruction, and other pathological changes, inducing or aggravating the occurrence and development of VD. There has been increasing evidence that the immune system plays a key role in brain-gut signal transduction (81). For example, studies in germ-free mice and mice treated with broad-spectrum antibiotics showed that the gut microbiota was involved in bacteria-related intestinal immunity, which also reflected the microbiota’s ability to locally regulate innate and adaptive immunity in the gastrointestinal tract (GI) and throughout the body (82). As we know, neurons and glia in the enteric nervous system are involved in intestinal immunity (83). Innate immune cells in the gut include dendritic cells, M cells, macrophages, mast cells, NK cells, and type 1, 2, and 3 innate lymphoid cells (ILCs), serving as the first line of defense in the intestinal barrier. Adaptive immune cells are divided into different cell subtypes (84). Cytotoxic CD8+T cells and CD4 effector cells (TH1 cells, TH2 cells, and Th17 cells) and regulatory T cells (Tregcells) are not only involved in action locally, but also migrate to other organs by immune migration, including the brain (85). Under normal conditions, the CNS is isolated from the periphery by the BBB, which is molecularly and cellularly similar to glial cells, and local immune functions are mainly supported by glial cells. Animal experiments had also demonstrated (86, 87) that when the BBB was damaged, its permeability increased, leading to microglia activation. Microglia are the main resident immune cells in the central nervous system, which have the ability of antigen presentation and bind to lymphocytes to limit the invasion of pathogens (88). In addition to cytokines, microglia can recognize neurotransmitters and neuromodulators, which trigger immune responses (88). As the most abundant glial cells in the nervous system, astrocytes play a key role in promoting the integrity of the BBB and participating in the immune response (89).

Blood ECs have long been known to modulate inflammation by regulating immune cell trafficking, activation status and function (90). ECs play a crucial role in BBB vascular biology by upholding permeability, homeostasis, vessel wall integrity, and preventing thrombosis (91), and are involved in peripheral immunity and neuroinflammation (92). In our study, we found that the functions of coDEGs are mainly concentrated in immune regulation and inflammatory response, and the dysfunction of these functions plays an important role in the permeability and function of ECs. For example, during sepsis, ECs enhance the immune response and activate the clotting system, thus shifting from an anticoagulant state to a pro-coagulant state (93, 94). They are both a target and source of inflammation, acting as a link between local and systemic immune responses. Inflammatory mediators also inhibited cAMP/Rac1signalling to increase endothelial cell permeability (95, 96). Severe inflammation can also result in parenchymal infiltration of peripheral immune cells and activation, causing structural damage to the BBB (97). When the BBB is damaged due to various reasons, the leaky BBB is involved in neuroinflammation, oxidative stress and excitotoxicity, leading to perivascular injury, leukocyte infiltration and brain parenchymal changes, leading to VD (98, 99). In addition, ECs can crosstalk with a variety of cells leading to cognitive impairment, for example, loss of endothelial-pericyte crosstalk is a major driving force in dementia pathology (100), and endothelium-macrophage crosstalk mediates BBB dysfunction in hypertension (101). Impaired BBB can cause a variety of neurological dysfunctions, which further leads to the imbalance of peripheral system homeostasis, leading to multiple intestinal complications, such as intestinal flora imbalance (GMD), IBD, necrotizing enterocolitis (NEC), and even colorectal cancer (CRC). In turn, these complications can worsen brain function and, in patients with IS, it can even disrupt the brain’s immune system (102).

Combined with the results of immune infiltration results of 8 hub genes, it can be inferred that intestinal diseases may migrate to the central nervous system through acquired immune cells such as Cytotoxic CD8+T cells, TH2 cells, and regulatory T cells to activate neural immunity. Of course, innate immune cells, especially NK cells, and mast cells are also directly involved in the formation of vascular dementia, which needs further experimental proof. Central memory CD4 T cells in VD and memory B cells in IBD can suppress this abnormal immune response and may provide targets for further intervention. Previous studies have found that intestinal immune cells themselves can also directly regulate neuroimmune homeostasis and brain responses to inflammation. Intestinal antigens stimulate B cells to differentiate into immunoglobulin A (IgA), secreting plasma cells to control the gut microbial community. Autoimmune diseases of the nervous system induce massive migration of intestinal IgA+ plasma cells to the brain and spinal cord (85). These findings are consistent with our results and may open new avenues for the treatment of nervous system disorders by using intestinal antigens to promote IgA+B cell production and promote neuroimmune suppression (103), and thus may provide potential immunotherapy targets for patients with IBD complicated with VD. In addition, evidence from animal studies suggested a potential role for short-chain fatty acids (SCFAs) in communication with the BBB and the brain-gut axis that supported BBB integrity (104), which could link butyric acid to memory, cognition, mood, and metabolism (105). Benakis et al. found that the intestinal flora of mice treated with antibiotics was changed, and the intestinal dysbiosis could increase regulatory T cells and decrease the number of IL-17+γδT cells by changing the activity of dendritic cells, and finally play a brain-protective role by limiting neuroinflammatory response (37). After middle cerebral artery occlusion, γδT cells would quickly infiltrate the lesion area and aggravate the BBB injury by releasing IL-17A.

Many scientists believe that there is a close link between microbial diversity and healthy aging. Studies in mice have shown that fecal microbiota grafts could correct age-related immune deficits (82), and that transplanting gut microbes from older mice into younger mice adversely affects critical CNS functions (106, 107). Neurological studies have shown that the microbiome also plays a role in neurodegeneration. Studies of fecal microbiota transplantation in transgenic mouse models indicated a causal relationship between gut microbiota, protein aggregation, and cognitive problems (108, 109). Animal and human clinical trials had explored dietary additives such as prebiotics, biostime, omega-3 polyunsaturated fatty acids, or phytochemicals to intervene in the intestinal microenvironment (110). For example, a high-fiber diet (which promoted SCFA production in the gut microbiota) was a promising intervention. It could overcome the cognitive and social dysfunction caused by maternal obesity (111). Fecal microbiota transplantation (FMT) was another potential treatment that had been shown to affect high-fat dietary intake in mice and reduce the risk of causing cognitive problems (112). Therefore, intestinal microbiome intervention is another promising target for improving cognitive impairment, which is consistent with the evidence of IBD and VD provided in this study.

This study has certain limitations. Initially, the data utilized in our research was sourced from the GEO database, which did not provide specific clinical details like gender, age, complications, etc. for each sample, resulting in their exclusion from our study. Additionally, the sample size derived from the database was limited, which might lead to false positive results. Moreover, the analysis of genes and pathways has certain limitations and cannot fully reflect the unknown mechanism of the disease. Consequently, further research with larger sample sizes is necessary to elucidate the precise relationship between VD and IBD and its various phenotypes. Finally, due to the difficulty in obtaining samples, we did not conduct *in vitro* experiments, particularly with regard to how these genes act on immune cells through which pathways and how they affect the occurrence and process of diseases.

## 5 Conclusion

This study elucidates the common pathogenesis of VD and IBD and the connection between the two through the brain-gut axis pathway at the molecular level, suggesting that IBD and VD are closely related, and IBD may be an independent risk factor for VD. In addition, the immunoinfiltration analysis of the coDEGs suggests that the two can interact through the innate and adaptive immune system, and the inflammatory response, which provides a new perspective for basic research and clinical research. The identification of 8 hub genes helps us to better search for new targets for the treatment of both, and is of great value for the prediction of IBD-combined VD.

## Data availability statement

The original data used in this study are all publicly available. This data can be found here: https://www.ncbi.nlm.nih.gov/geo/ under the accession numbers GSE122063, GSE47908, and GSE479285.

## Ethics statement

Ethical approval was not required for the studies involving humans because All data in this paper are from public databases. The studies were conducted in accordance with the local legislation and institutional requirements. The human samples used in this study were acquired from gifted from another research group. Written informed consent to participate in this study was not required from the participants or the participants’ legal guardians/next of kin in accordance with the national legislation and the institutional requirements.

## Author contributions

YW: Data curation, Formal analysis, Investigation, Visualization, Writing – original draft. DX: Conceptualization, Funding acquisition, Writing – review & editing. SM: Data curation, Methodology, Validation, Writing – review & editing. NS: Project administration, Resources, Writing – review & editing. XZ: Project administration, Software, Writing – review & editing. XW: Supervision, Validation, Writing – review & editing.
