# Supplementary figures and images for: Exploring the common mechanism of vascular dementia and inflammatory bowel disease: a bioinformatics-based study
Source: Front Immunol. 2024 Apr 25;15:1347415. doi: 10.3389/fimmu.2024.1347415 (PMC11084673; doi:10.3389/fimmu.2024.1347415)

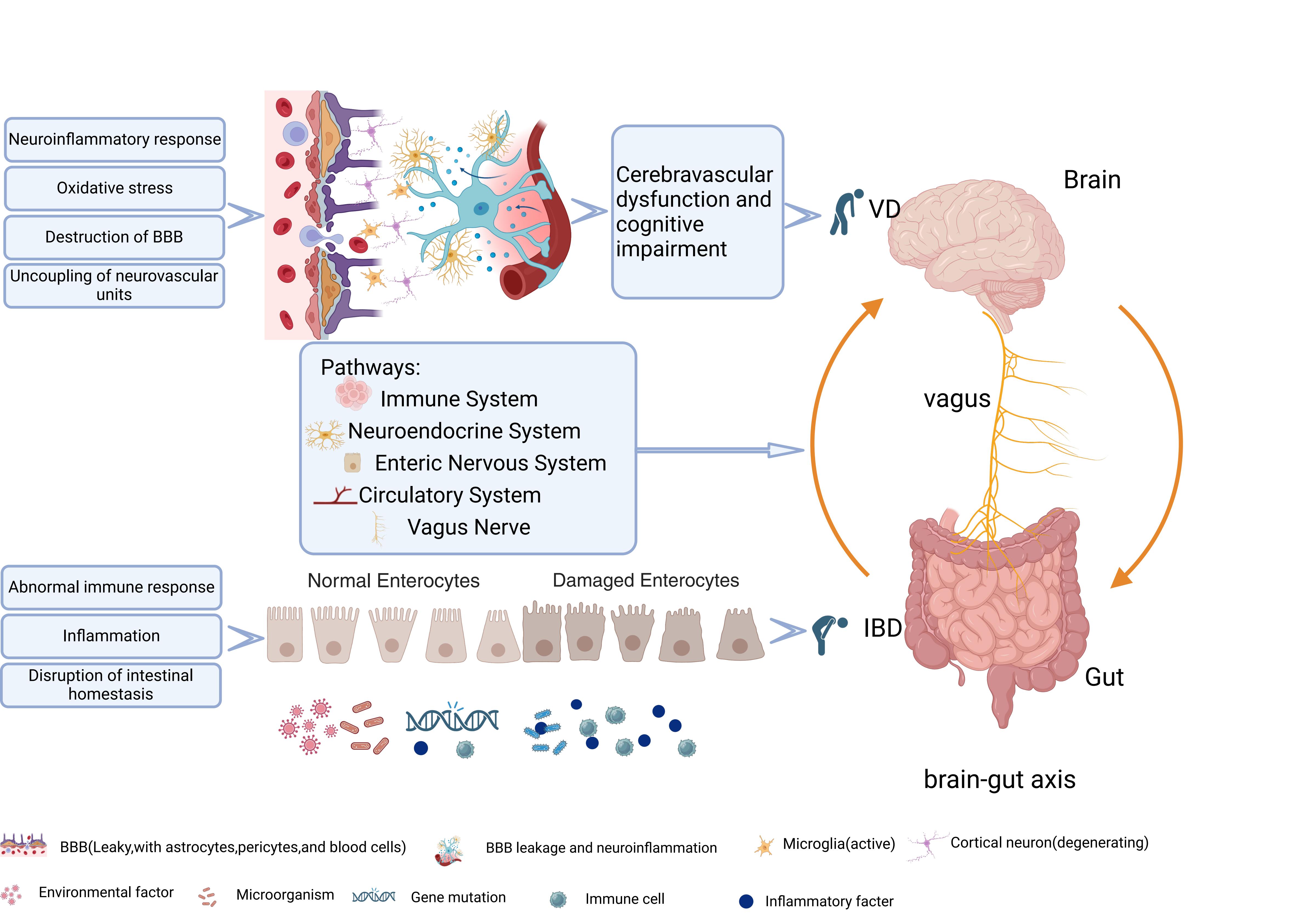

Supplement: Supplementary file 1 [file DataSheet_1.zip › Supplementary material/FIGURE 1-11/FIGURE 1.jpg]

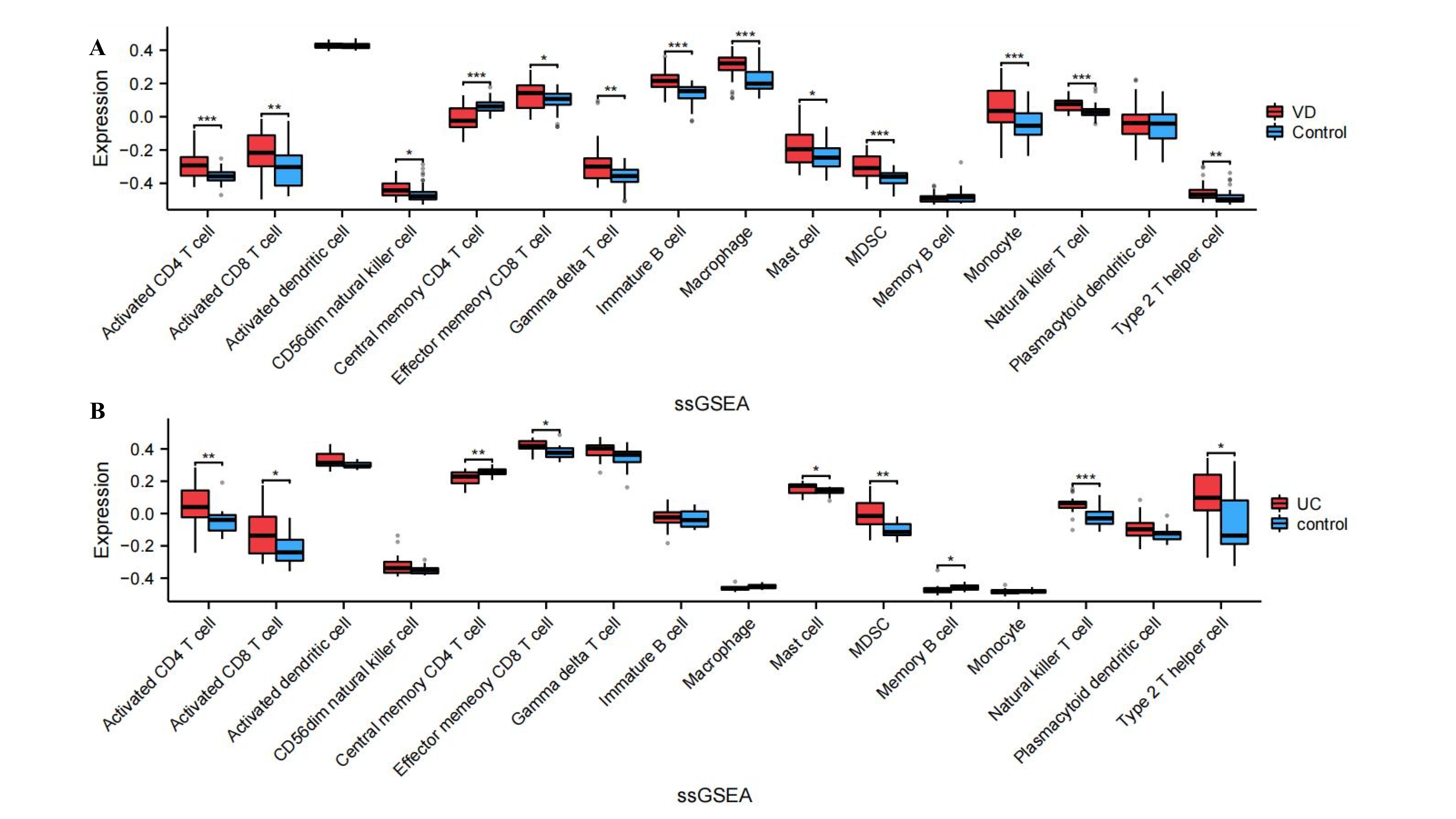

Supplement: Supplementary file 1 [file DataSheet_1.zip › Supplementary material/FIGURE 1-11/FIGURE 10.jpg]

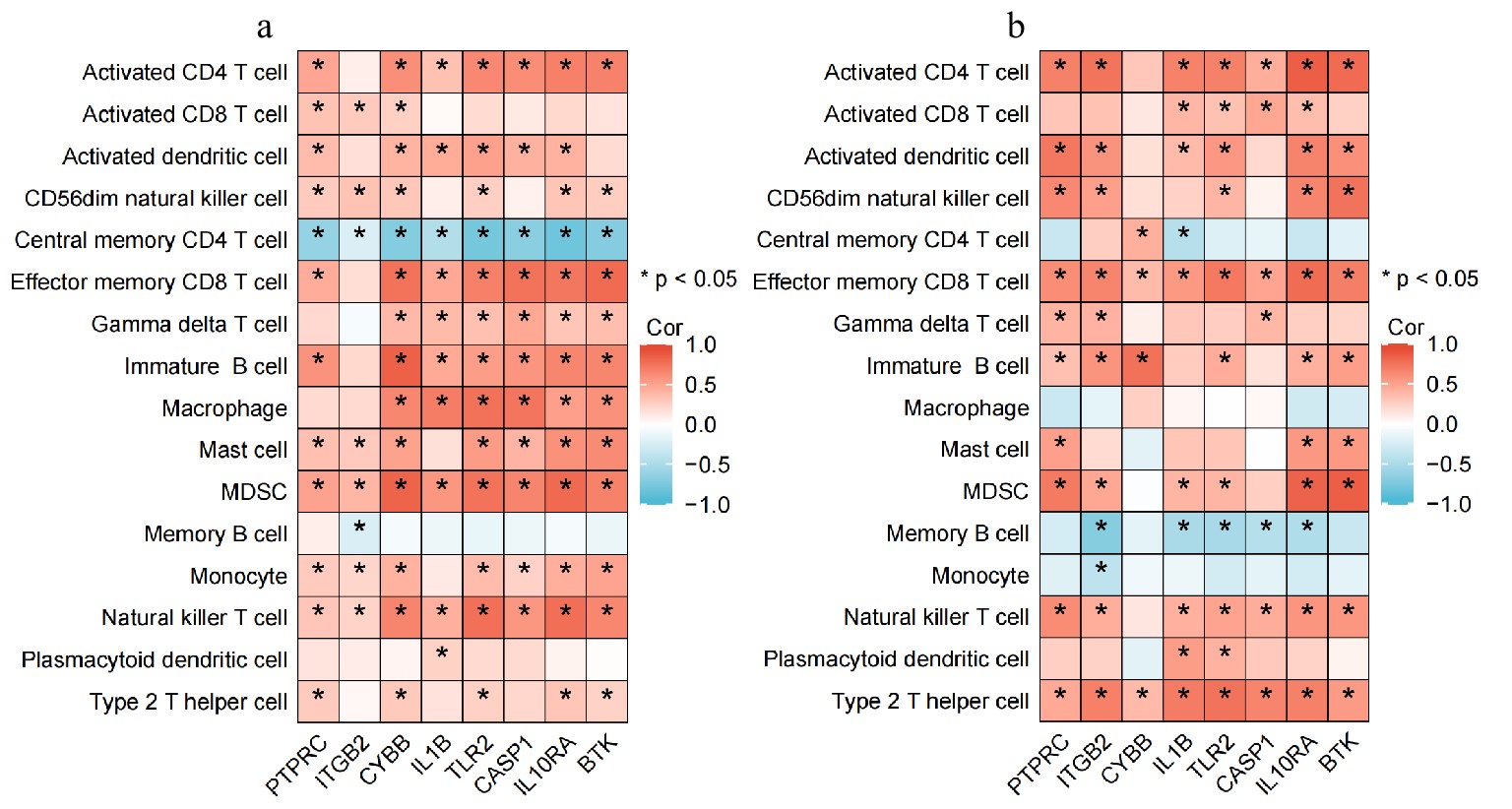

Supplement: Supplementary file 1 [file DataSheet_1.zip › Supplementary material/FIGURE 1-11/FIGURE 11.jpg]

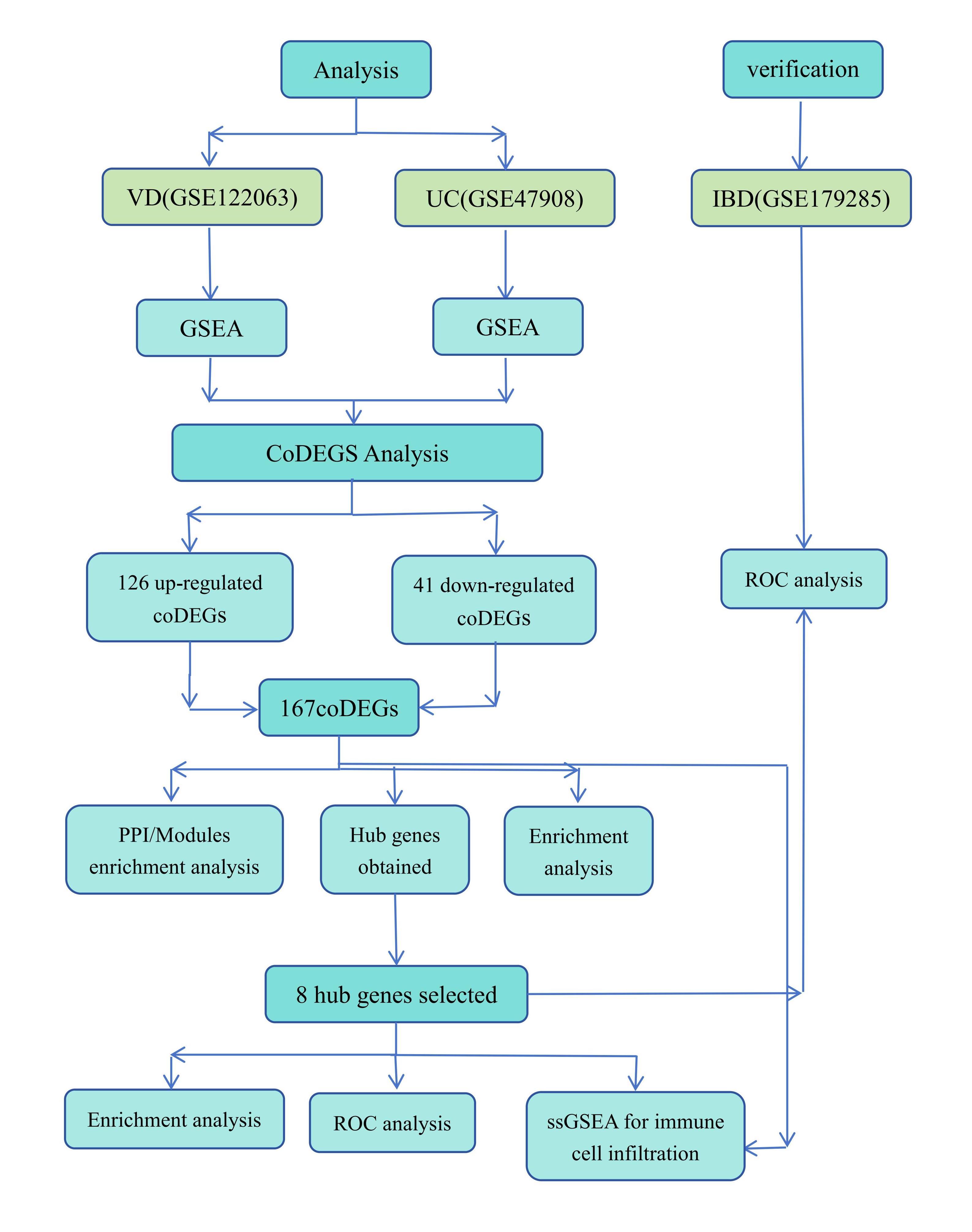

Supplement: Supplementary file 1 [file DataSheet_1.zip › Supplementary material/FIGURE 1-11/FIGURE 2.jpg]

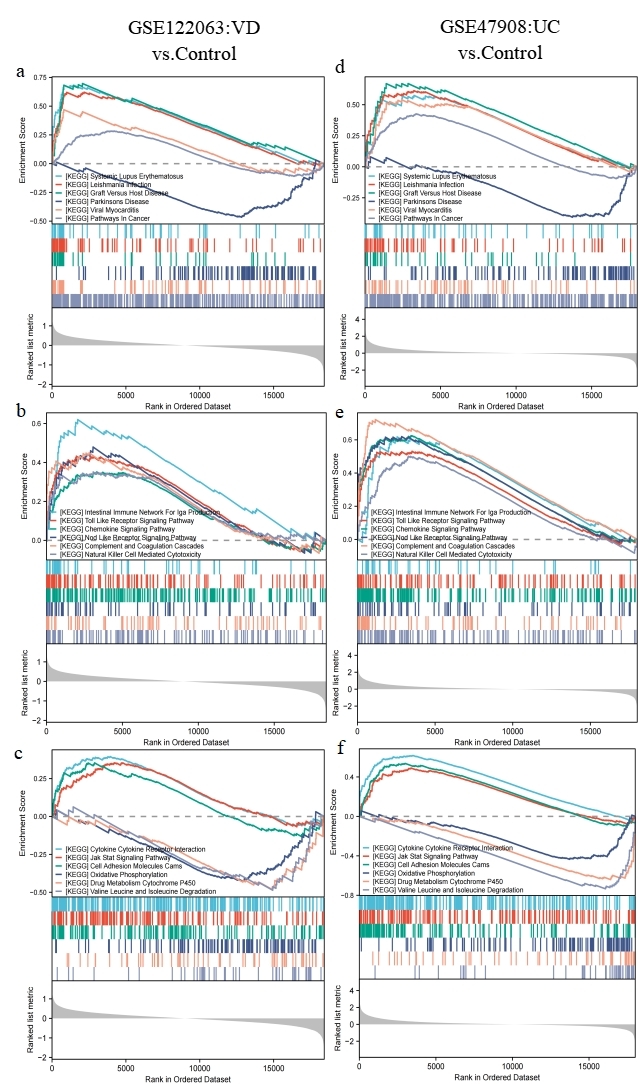

Supplement: Supplementary file 1 [file DataSheet_1.zip › Supplementary material/FIGURE 1-11/FIGURE 3.jpg]

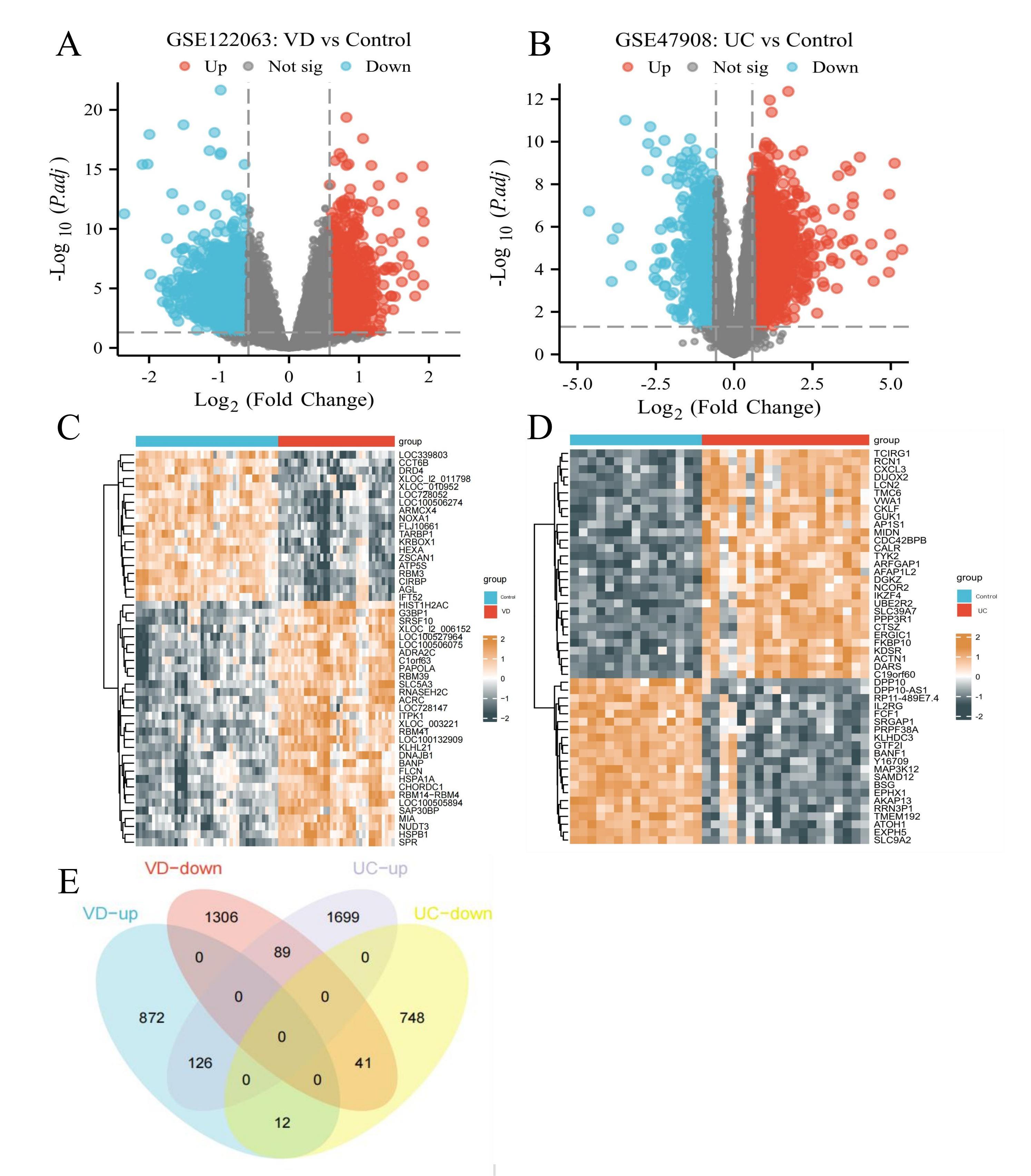

Supplement: Supplementary file 1 [file DataSheet_1.zip › Supplementary material/FIGURE 1-11/FIGURE 4.jpg]

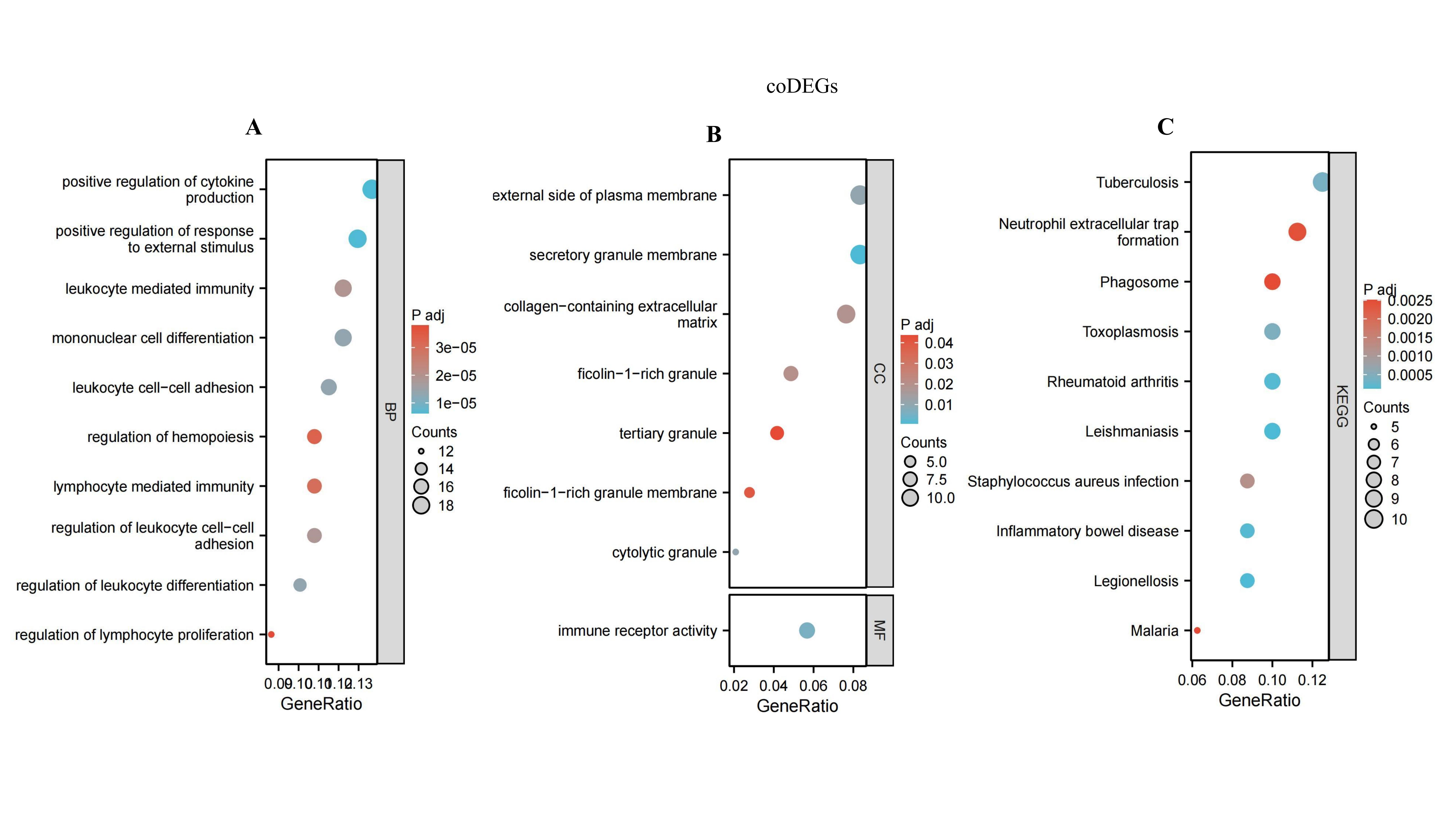

Supplement: Supplementary file 1 [file DataSheet_1.zip › Supplementary material/FIGURE 1-11/FIGURE 5.jpg]

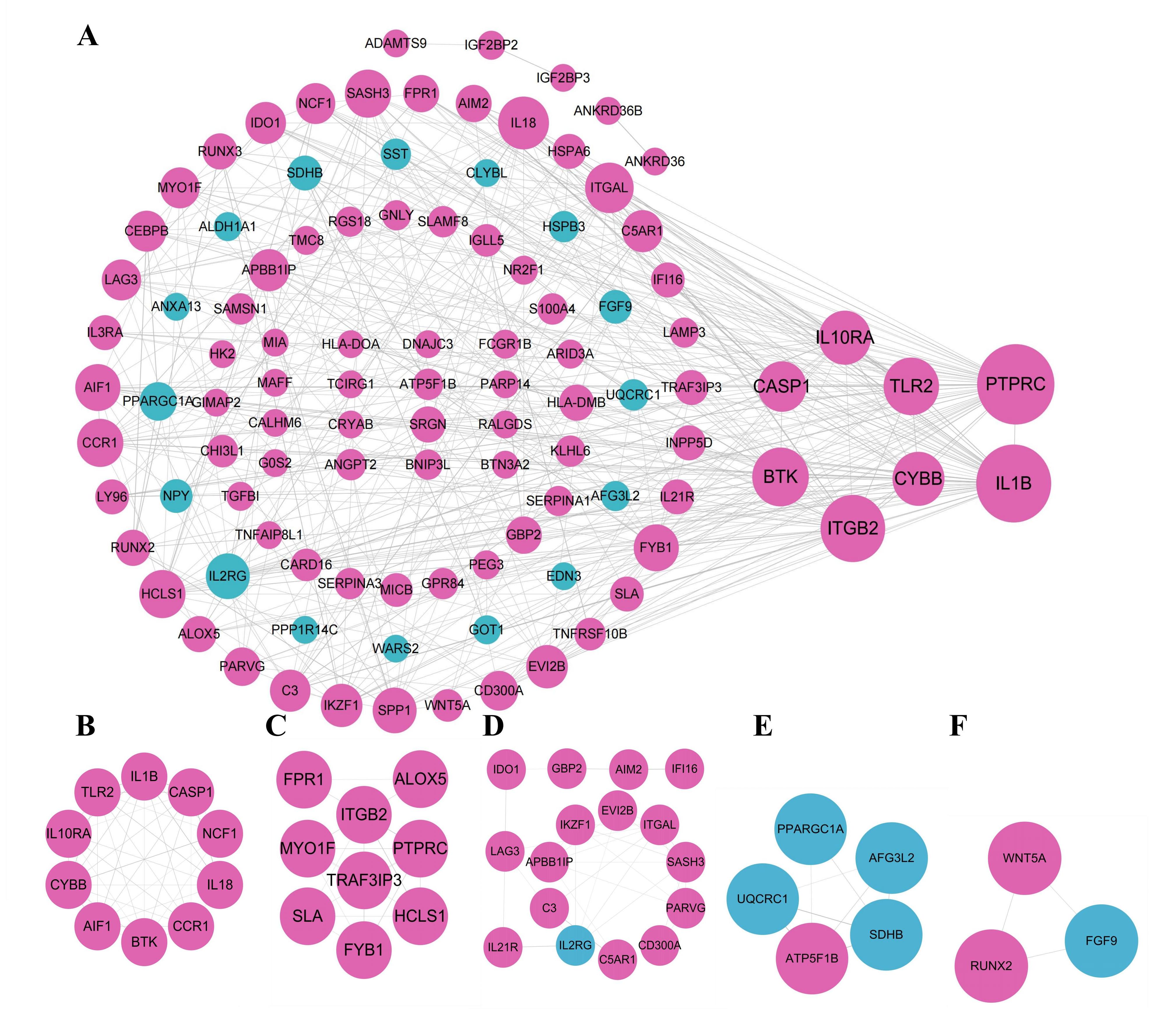

Supplement: Supplementary file 1 [file DataSheet_1.zip › Supplementary material/FIGURE 1-11/FIGURE 6.jpg]

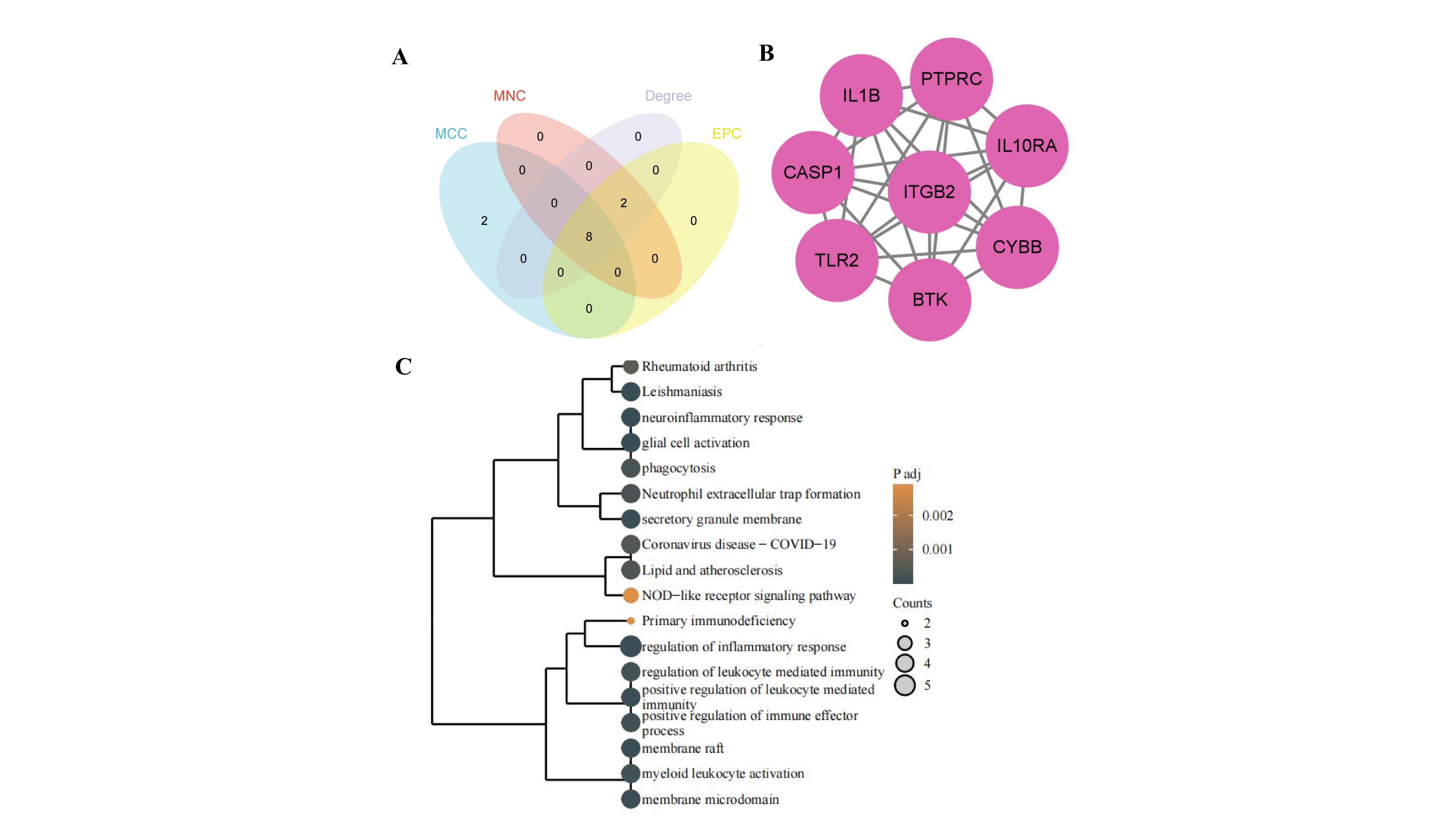

Supplement: Supplementary file 1 [file DataSheet_1.zip › Supplementary material/FIGURE 1-11/FIGURE 7.jpg]

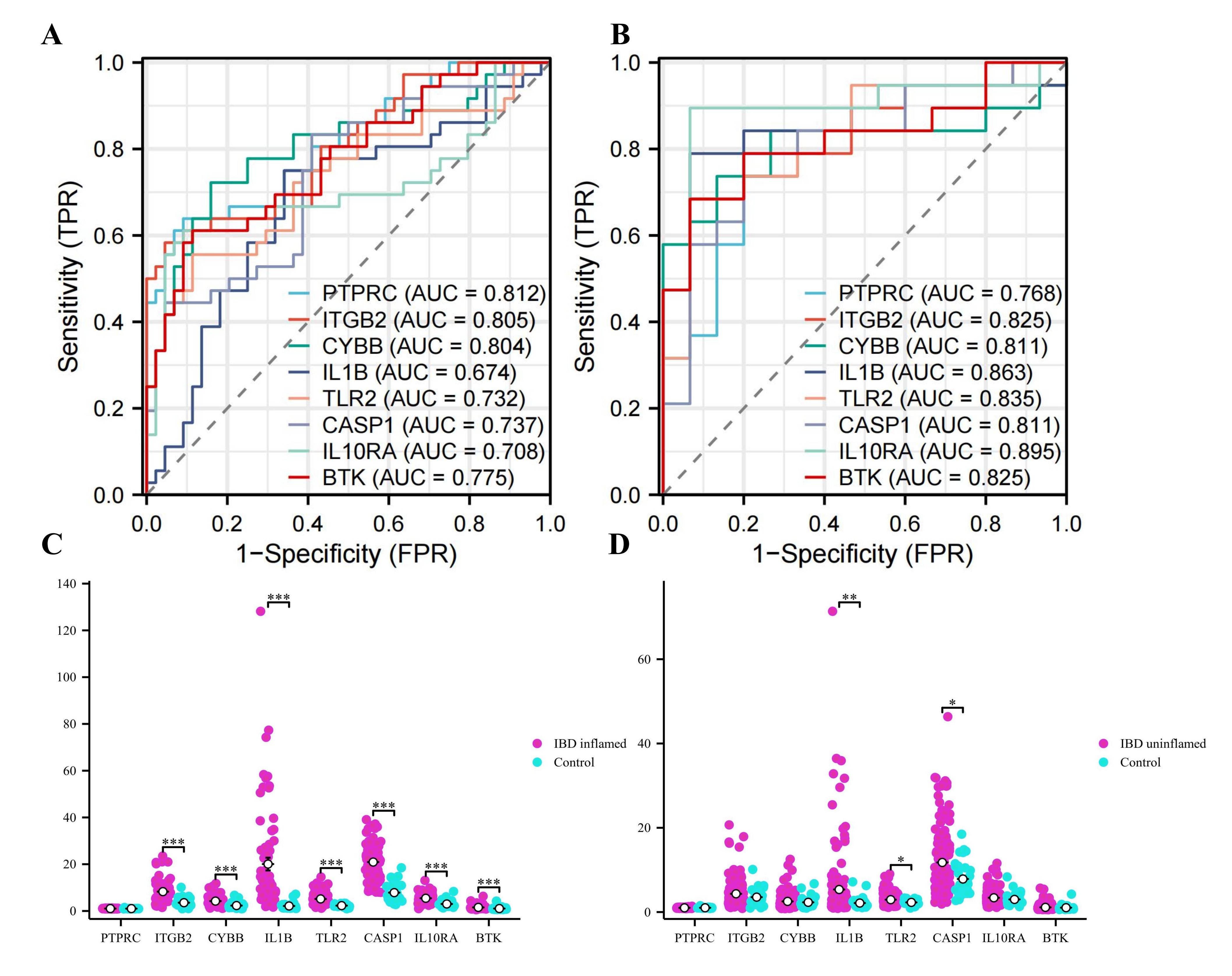

Supplement: Supplementary file 1 [file DataSheet_1.zip › Supplementary material/FIGURE 1-11/FIGURE 8.jpg]

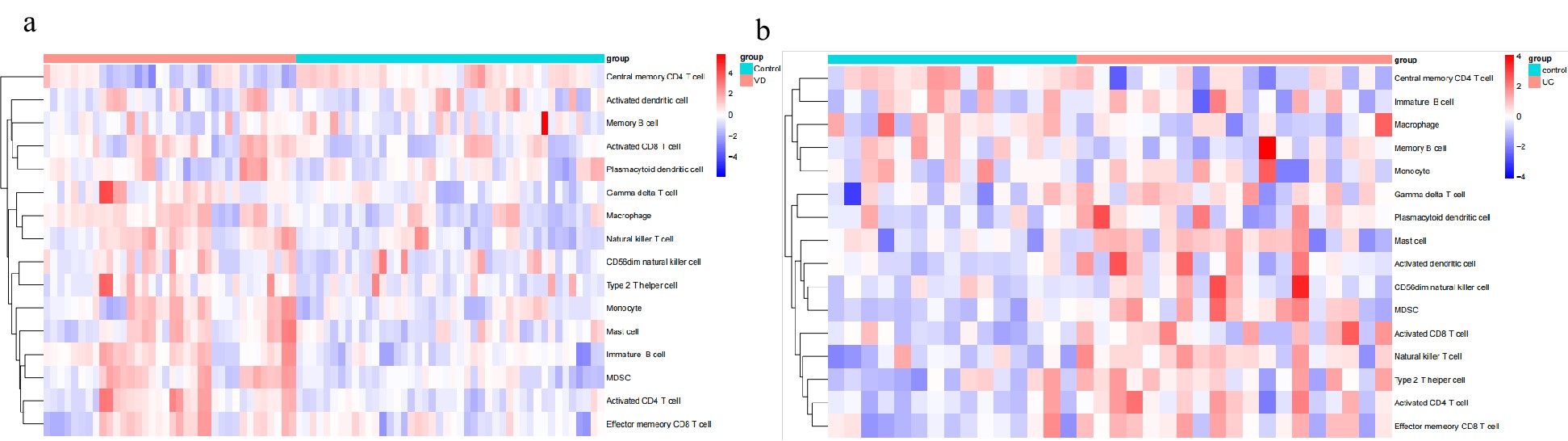

Supplement: Supplementary file 1 [file DataSheet_1.zip › Supplementary material/FIGURE 1-11/FIGURE 9.jpg]

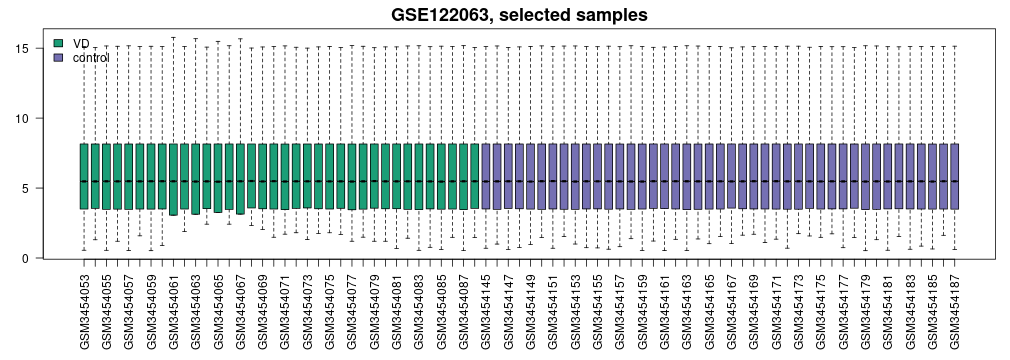

Supplement: Supplementary file 1 [file DataSheet_1.zip › Supplementary material/other FIGURES/GSE122063 Graph of normalization.jpg]

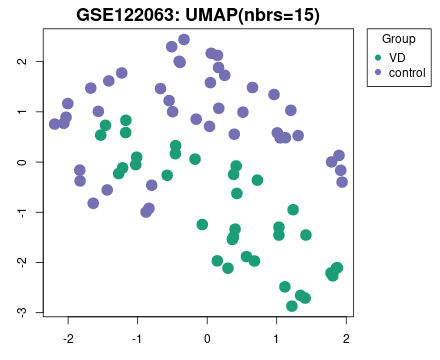

Supplement: Supplementary file 1 [file DataSheet_1.zip › Supplementary material/other FIGURES/GSE122063 UMAP.jpg]

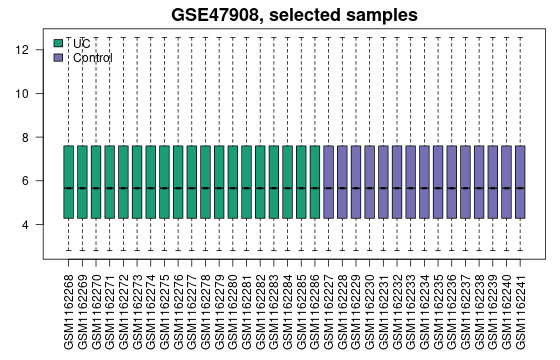

Supplement: Supplementary file 1 [file DataSheet_1.zip › Supplementary material/other FIGURES/GSE47908 Graph of normalization.jpg]

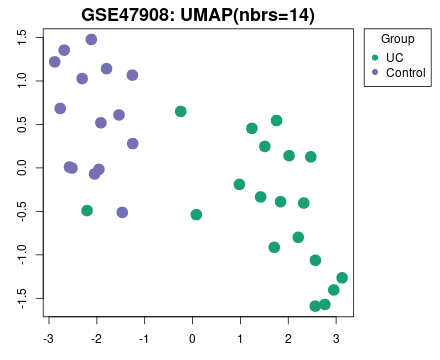

Supplement: Supplementary file 1 [file DataSheet_1.zip › Supplementary material/other FIGURES/GSE47908 UMAP.jpg]
